# Molecular effects of resistance elicitors from biological origin and their potential for crop protection

**DOI:** 10.3389/fpls.2014.00655

**Published:** 2014-11-21

**Authors:** Lea Wiesel, Adrian C. Newton, Ian Elliott, David Booty, Eleanor M. Gilroy, Paul R. J. Birch, Ingo Hein

**Affiliations:** ^1^Cell and Molecular Sciences, The James Hutton InstituteDundee, UK; ^2^OMEX Agriculture Ltd.Lincoln, UK; ^3^The Division of Plant Sciences, College of Life Science, University of Dundee at the James Hutton InstituteDundee, UK

**Keywords:** crop protection, elicitors, pathogen effectors, priming, disease resistance

## Abstract

Plants contain a sophisticated innate immune network to prevent pathogenic microbes from gaining access to nutrients and from colonizing internal structures. The first layer of inducible response is governed by the plant following the perception of microbe- or modified plant-derived molecules. As the perception of these molecules results in a plant response that can provide efficient resistance toward non-adapted pathogens they can also be described as “defense elicitors.” In compatible plant/microbe interactions, adapted microorganisms have means to avoid or disable this resistance response and promote virulence. However, this requires a detailed spatial and temporal response from the invading pathogens. In agricultural practice, treating plants with isolated defense elicitors in the absence of pathogens can promote plant resistance by uncoupling defense activation from the effects of pathogen virulence determinants. The plant responses to plant, bacterial, oomycete, or fungal-derived elicitors are not, in all cases, universal and need elucidating prior to the application in agriculture. This review provides an overview of currently known elicitors of biological rather than synthetic origin and places their activity into a molecular context.

## The role of defense elicitors in plant immunity

Plants are under constant threat of microbial pathogen attack. Plant cell walls, cuticles and phytoanticipins are preformed, physical and chemical barriers that limit access of microbes to plant cells (Underwood, 2012; Newman et al., 2013). In addition to these non-inducible defenses, plants recognize and respond to defense elicitors which are signal-inducing compounds perceived by the innate immune system that prime and/or induce defense responses (Henry et al., 2012; Maffei et al., 2012; Newman et al., 2013). Elicitor compounds can be biological in origin, derived from either the plant or the microbe, or can be synthetically generated (Walters et al., 2013). We will focus on elicitors from biological origin (Table 1) rather than synthetic analogs of known signaling or defense molecules such as Bion, acibenzolar-S-methyl (ASM), beta-amino-butyric acid (BABA), and cis-jasmone. Elicitor activity has, for example, been shown for plant-derived cell wall components such as oligogalacturonides (Ferrari et al., 2013), proteinaceous pathogen molecules such as bacterial flagellin (Gomez-Gomez and Boller, 2002), oomycete-derived elicitin INF1 (reviewed in Hein et al., 2009) and non-proteinaceous molecules such as lipopolysaccharides (Erbs and Newman, 2012). However, intact plant- or microbe-derived structures as well as highly-polymerized molecules often tend to result in few recognition responses. In contrast, leakage of metabolites or even minor or partial breakdown of complex host or pathogen molecules leads to the production of eliciting components that are biologically active.

**Table 1:** **List of plant-, bacterial-, oomycete-, and fungal-derived elicitor compounds, their activity against pathogens and effectiveness in plants**.

**Origin**	**Elicitor compound**	**Effective toward**	**Plants effects shown in**	**References**
Plant	Oligogalacturonides	*Botrytis cinerea, Blumeria graminis*	Several	Aziz et al., 2007; Randoux et al., 2010; Galletti et al., 2011
	Milsana (giant knotweed)	*Botrytis cinerea, Leveillula taurica*	Cucumber, tomato	Daayf et al., 1997, 2000; Konstantinidou-Doltsinis et al., 2006
	Burdock fructooligosaccharide	*Colletotrichum lagenarium, Botrytis cinerea*, TMV	Cucumber, tobacco, tomato	Wang et al., 2009; Guo et al., 2012
	Elicitor peptide 1 (Pep1)	*Cochliobolis heterostrophus*, *Colletotrichum graminicola*	Maize	Huffaker et al., 2011
	Carrageenans	*Sclerotinia sclerotiorum*, TMV	*A. thaliana*, tobacco	Sangha et al., 2010; Vera et al., 2011
	Fucans	TMV	Tobacco	Vera et al., 2011
	Ulvans	Several	Several	Jaulneau et al., 2011; Vera et al., 2011
	Laminarin	*Erwinia carotovora, Plasmopara viticola, Botrytis cinerea*, *Fusarium solani*	Beans, grapevine, tobacco	Craigie, 2011; Vera et al., 2011
Bacteria	Harpin	*Xanthomonas oryzae*	Rice	Lee et al., 2001; Li et al., 2012
	Lipopeptides	*Botrytis cinerea*	Tomato	Henry et al., 2012
	Dimethylsulfide	*Cochliobolus heterostrophus, Botrytis cinerea*	Maize, tobacco	Huang et al., 2012
	Pseudobactin	*Botrytis cinerea, Erwinia carotovora*	Several	De Vleesschauwer and Höfte, 2009
Oomycetes	CBEL	*Phytophthora parasitica*	*A. thaliana*, tobacco	Mateos et al., 1997; Khatib et al., 2004
	Cryptogein	*Phytophthora parasitica, Sclerotinia sclerotiorum*	Tobacco	Bonnet et al., 1996
	Eicosapentaenoic acid	*Phytophthora infestans*	Potato	Henriquez et al., 2012
	Pep-13	*Phytophthora* spp.	Parsley, potato	Nürnberger et al., 1994; Brunner et al., 2002; Parker, 2003
	INF1	*Phytophthora infestans*	Tobacco	Takahashi et al., 2007; Hein et al., 2009; Kawamura et al., 2009
Fungi	β-glucans	Several	Several	Hahn and Albersheim, 1978; Fu et al., 2011; Falcón-Rodríguez et al., 2012; Henriquez et al., 2012
	Chitosan	Several	Several	Kishimoto et al., 2010; Kombrink et al., 2011
	Chitin	Several	Several	El Ghaouth et al., 1994; Copping and Duke, 2007; El Hadrami et al., 2012
	Ergosterol	*Botrytis cinerea*	Grapevine, tobacco	Laquitaine et al., 2006; Vatsa et al., 2011
	*Trichoderma species*: xylanases, peptaibol, cerato-platanin family	*Pseudomonas syringae, Botrytis cinerea, Colletotrichum graminicola*	*A. thaliana*, cotton, maize	Ron and Avni, 2004; Djonoviç et al., 2007; Viterbo et al., 2007; Yang et al., 2009; de Oliveira et al., 2011
	Cerebrosides	*Fusarium* spp.	Several	Umemura et al., 2004
	HR-inducing protein	*Magnaporthe oryzae*	Rice	Chen et al., 2012; Kulye et al., 2012
	PeaT1	TMV	Tobacco	Zhang et al., 2010, 2011b
	PebC1	*Botrytis cinerea*	Tomato	Zhang et al., 2010
	PevD1	TMV	Tobacco	Wang et al., 2012a,b
	PemG1	*Pseudomonas syringae, Xanthomonas oryzae*	*A. thaliana*, rice	Qiu et al., 2009; Peng et al., 2011

**Figure 1:**
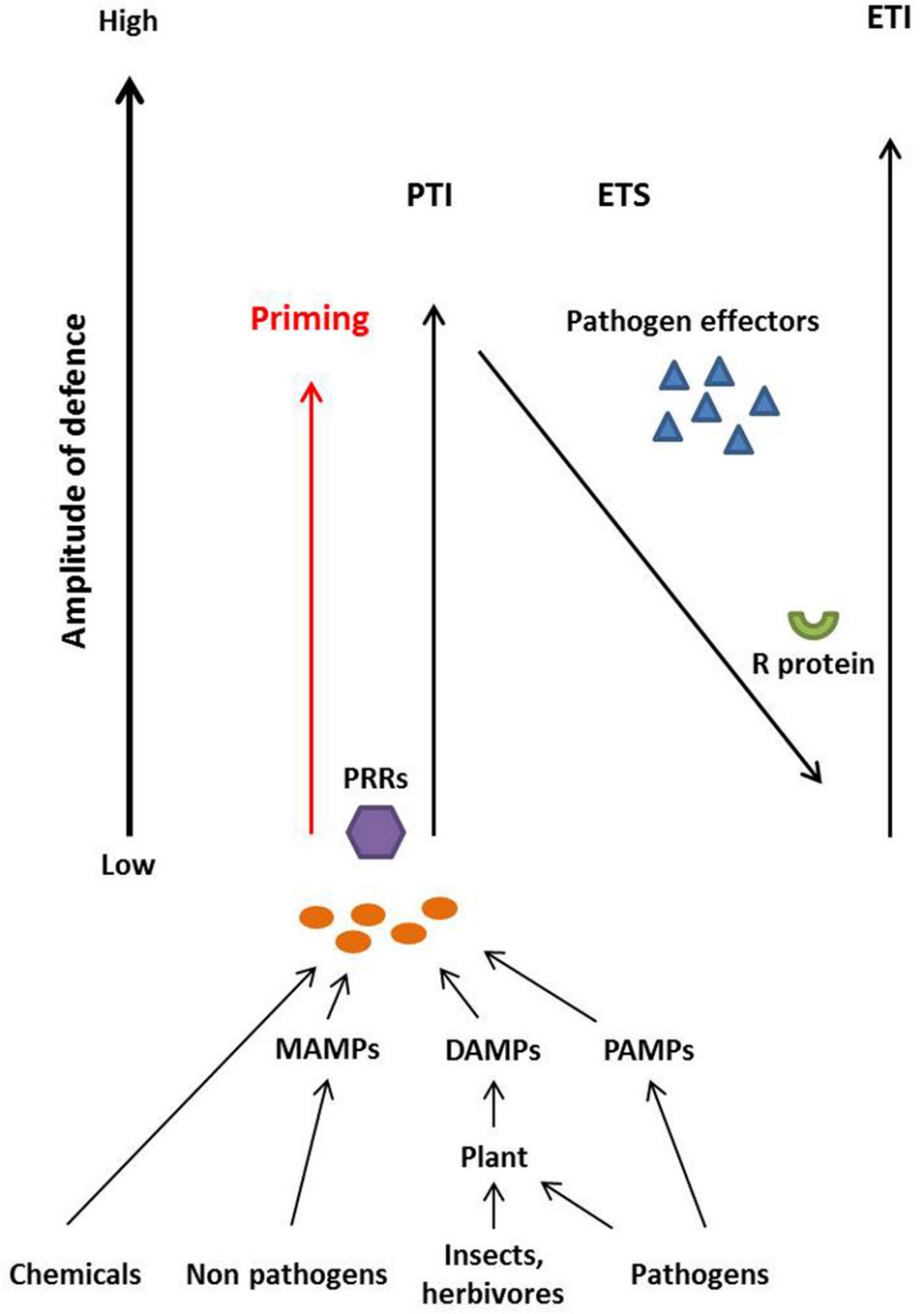
**Plants recognize chemical elicitors, Microbe-Associated Molecular Patterns (MAMPS) derived from non-pathogenic microbes, Pathogen-Associated Molecular Patterns (PAMPS) derived from pathogens and Damage-Associated Molecular Patterns (DAMPS) that are produced by plants upon insect, herbivore or pathogen attack, via transmembrane Pattern Recognition Receptors (PRRs)**. The recognition leads to the onset of defense mechanisms referred to as pattern-triggered immunity (PTI). Adapted pathogens secrete effectors that disturb plant defense mechanisms leading to effector-triggered susceptibility (ETS). Plant resistance (R) proteins recognize pathogen effectors and induce effector-triggered immunity (ETI). Treatment of plants with elicitor compounds (chemicals, MAMPs, DAMPs, or PAMPs) in the absence of adapted pathogen leads to priming and/or PTI-based immunity that put plants into an alerted stage of defense that provides some enhanced resistance toward otherwise virulent pathogens. Figure adapted from Henry et al. (2012), and Jones and Dangl (2006).

The co-evolution between plants and potential microbial pathogens has been described as a zigzag model by Jones and Dangl (2006) and can also be applied to deducing the biological activity of elicitors (Figure 1). According to the zigzag model, the first inducible responses are a consequence of the perception of chemical elicitors, microbe-associated molecular patterns (MAMPs), pathogen-associated molecular patterns (PAMPs) and/or damage-associated molecular patterns (DAMPs). The latter are also known as danger-associated molecular patterns (Mazzotta and Kemmerling, 2011). MAMPs describe general microbe-derived molecules including those originating from beneficial microbes whereas PAMPs specifically describe molecules from pathogenic microbes such as fungi, oomycetes, and bacteria (Henry et al., 2012; Newman et al., 2013). Thus, PAMPS are a subgroup of MAMPs (Maffei et al., 2012). In contrast, DAMPs are typically plant-derived and are produced after, for example, wounding by insects or herbivores as well as degradation or perturbation of host molecules by microbes (Henry et al., 2012; Newman et al., 2013). All of these molecules, which could universally be described as “patterns that elicit immunity” (PEIs), are often recognized by transmembrane pattern recognition receptors (PRRs) in plant cells (Jones and Dangl, 2006; Maffei et al., 2012; Newman et al., 2013). Upon recognition of MAMP- or DAMP-derived patterns, PTI (PAMP- or pattern-triggered immunity) is activated in the plant and the perceived molecules could be described as immune elicitors. This defense reaction aims to restrict the growth of the intruder and can lead to systemic induced resistance leaving the plant less susceptible to subsequent pathogen attack (Henry et al., 2012).

Systemic induced resistance can be divided into systemic acquired resistance (SAR) or induced systemic resistance (ISR). Systemic acquired resistance is often characterized by localized necrosis, expression of pathogenesis related (PR) protein genes, and involves the salicylic acid (SA) pathway whereas ISR is often triggered by plant growth-promoting rhizobacteria (PGPR) (Walters et al., 2013), is not associated with necrosis and involves the jasmonic acid (JA) and ethylene (ET) pathways (Walters and Heil, 2007; Henry et al., 2012). Typical responses of PTI include cell wall alterations and the production of reactive oxygen species (ROS) which can be directly cytotoxic but also play a role in signaling. Other responses comprise the production of phytoalexins, expression of PR proteins, activation of mitogen activated protein kinase (MAPK) pathways, and defense signaling involving calcium (Ca^2+^) influx from extracellular spaces and changes in free cytosolic Ca^2+^ concentrations (Garcion et al., 2007). To counteract the initial plant defense reaction, successful microbes have evolved specialized effectors that perturb recognition of defense elicitors or subsequent plant defense mechanisms to promote effector-triggered susceptibility (ETS). However, if these pathogen effectors are in turn recognized by cognate plant resistance (R) proteins, the second layer of inducible response, effector-triggered immunity (ETI), is initiated that often yields a hypersensitive resistance response (HR) (Jones and Dangl, 2006; Deslandes and Rivas, 2012).

The outcome of plant/microbe interactions can result in symbiosis, disease or disease resistance and is governed by further levels of sophisticated co-evolution. Indeed, it must be recognized that pathogen colonization of plants can generate dynamic pathogenic, mutualistic or parasitic interactions of varying magnitude and specificity. Furthermore, organisms recognized as pathogens in, for example, a crop context, could be benign or even beneficial in another context such as a different host or environment (Newton et al., 2010). It is thus essential for the plant to evaluate the scale of threat and to mount appropriate and proportionate responses. These may range from priming, being ready to respond faster to actual attack, or expression of PTI-based defense mechanisms to yield incompatibility if the microbe/pathogen is unable to suppress these responses. The use of elicitors in agriculture holds the potential to decrease the need for pesticide application by using the plant's own defense system. However, there is a need to understand this process on a molecular level to maximize the efficacy of the treatments.

## Inducible defense response in the absence of pathogens

In agricultural practice, elicitor treatments of plants in the absence of virulent pathogens yields a defense response such as priming and/or PTI that is uncoupled from ETS and can provide some protection to subsequent pathogen challenges. Priming is defined as a physiological status of plants leading to faster and stronger activation of defense responses to subsequent biotic and abiotic stresses (reviewed in Conrath et al., 2006; Conrath, 2011; Pastor et al., 2013). Crucially, this is distinct from the level of resistance induction in response to the recognition of true pathogens that are potentially capable of causing disease and where recognition would cause resistance mechanism expression that is more costly to the plant but still proportionate to potential disease cost (Walters and Heil, 2007).

In primed plants, chromatin modifications in the form of methylation and acetylation of histones take place that impact on the interaction of DNA with histones and/or open binding sites for transcriptional co-activators such as WRKY22 and WRKY29 (Eulgem, 2005; Conrath, 2011; Po-Wen et al., 2013). These chromatin modifications in primed plants have been shown to lead to increased expression of transcription factors WRKY6, WRKY29, and WRKY53 after stress exposure (Jaskiewicz et al., 2011). In *Arabidopsis thaliana*, mRNA and inactive MPK3 and MPK6 accumulate in cells of elicitor-treated plants. Upon exposure to *Pseudomonas syringae* both MAP kinases are more strongly activated in primed plants than in non-primed plants (Beckers et al., 2009).

When PTI-associated mechanisms are primed by elicitor treatments plants often accumulate ROS and produce a stronger, secondary oxidative burst following pathogen challenge, activate MPKs and stimulate SA-, JA-, and abscisic acid (ABA)-pathways (Beckers et al., 2009; Pastor et al., 2013). Callose deposition, which is potentially also linked to the ABA-pathway, can be enhanced in elicitor-treated plants (Kohler et al., 2002; Flors et al., 2005; Pastor et al., 2013) and elicitor treatment often induces expression of phenylalanine ammonia lyase (PAL) which is required for the production of SA precursors (Chen et al., 2009). In line with SA involvement, pathogenesis-related genes such as PR-1, PR-2, and PR-5 have been implicated with elicitor treatments (Kohler et al., 2002; Conrath et al., 2006). Both priming and the activation of defense mechanisms due to elicitor treatment can lead to a reduction of disease severity when subsequent pathogen attack occurs. Biologically active defense elicitors that either prime plant defenses or initiate PTI responses have been identified from diverse sources. Molecular studies have provided clues to their mechanism and to the processes that govern specificity.

## A molecular perspective of elicitor activity in plant immunity

Several studies have shown that elicitor-treated plants show lower infection rates following inoculation with virulent pathogens but responses can vary between plant species (Table 1). In addition to the observed disease reduction, molecular studies are revealing how the elicitor compounds affect gene expression levels in plants and therefore impact on defense responses (Section Plant Genes and Pathways Involved in Elicitor Recognition). Similarly, the diverse mechanisms by which pathogen effectors suppress PTI responses are emerging but, due to the complexity of this research, only selected examples are highlighted in this review.

### Plant-derived elicitors

Plant cell walls are composed of cellulose, hemicellulose (cross-linking glycans), pectic polysaccharides, protein, lignin, and a variety of lipids (Wei et al., 2009). Bacteria and fungi can produce cellulases, xylanases, and lignin peroxidases that break down plant cell wall components and common products are β-glucans, xylose, and phenylpropanoid-containing compounds. These break-down products function as plant-derived elicitors and several examples of disease reduction due to the application of plant-derived elicitors exist (Table 1). Well studied plant-derived elicitors include oligogalacturonides (OGs), which are structural components of plant cell walls and are released upon partial degradation of homogalacturonan by microbial polygalacturonases during infection or by plant polygalacturonases induced upon wounding (Ferrari et al., 2013). Plant cell wall-derived OGs are recognized by wall-associated kinase 1 (WAK1) and subsequent signaling is JA-, SA-, and ET-independent (Brutus et al., 2010; Ferrari et al., 2013). A MAP kinase cascade is triggered upon OG perception in *A. thaliana*, and MPK3 and MPK6 are phosphorylated. However, the importance of these signaling events remains elusive and it has been shown, for example, that lack of MPK3 increases basal susceptibility to *Botrytis cinerea* but elicitor-induced resistances are not affected (Galletti et al., 2011). In contrast, MPK6 is necessary for OG-induced resistance but does not play a role in basal resistance toward *B. cinerea* (Galletti et al., 2011).

### Bacterial-derived elicitors

In addition to plant-derived elicitors, the application of bacterial-derived elicitors has also been shown to reduce pathogen infection in plants (Table 1). Extracellular polysaccharides (EPS) produced by the bacterial wilt causing pathogen *Ralstonia solanacearum* have been shown to induce defense responses in tomato (Milling et al., 2011) and lipopolysaccharides (LPS) from Gram-negative bacteria also trigger induced resistance in several other plant species (Dow et al., 2000; Gerber et al., 2004; Desaki et al., 2006; Erbs and Newman, 2012). PGPRs (plant growth-promoting rhizobacteria) can induce resistance in plants by exudating elicitors (De Vleesschauwer and Höfte, 2009 and references therein) and filtrates from cultures of bacteria such as *Bacillus subtilis* can also elicit crop protection effects (Schönbeck et al., 1980, 1982), though these may be a combination of direct toxicity and/or elicitor recognition events.

The molecular background to bacterial MAMPs, effectors and their plant targets has been reviewed recently (Deslandes and Rivas, 2012) and two well-studied bacterial MAMPs are flagellin and the elongation factor Tu (EF-Tu). Flagellin is recognized in a variety of plant species whereas EF-Tu, one of the most abundant proteins in bacterial cells, and bacterial cold-shock proteins seem to be specifically recognized in *Brassicaceae* and *Solanaceae* plants, respectively (Gomez-Gomez and Boller, 2002; Felix and Boller, 2003; Bittel and Robatzek, 2007; Jeworutzki et al., 2010). In both proteins, the N-terminus contains the eliciting site which, for flagellin, can often be described as a 22 amino acid long epitope (flg22), whereas that for EF-Tu is 18 amino acids (Elf18). Flagellin and EF-Tu are recognized by two distinct plant receptors (Gomez-Gomez and Boller, 2002; Kunze et al., 2004). Flagellin is recognized by FLAGELLIN-SENSING 2 (FLS2) whereas EF-Tu is recognized by EF-Tu RECEPTOR (EFR) which has only been found in Brassicaceae (Gomez-Gomez and Boller, 2002; Zipfel et al., 2006). This provides molecular insight into the specificity of elicitors and emphasizes the need to assess candidate defense eliciting compounds in a diverse range of plant species. It is interesting to note that heterologous expression of EFR in *Solanaceae* plants provides some resistance to bacteria that express EF-Tu (Lacombe et al., 2010) which suggests that downstream signaling cascades could be conserved for different PRRs and in different plant species. Indeed, both FLS2 and EFR are leucine rich repeat receptor like kinases (LRR-RLK) and both interact with BRI1-associated receptor kinase 1 (BAK1) triggering SA-, JA-, and ET-independent signaling (Zipfel et al., 2004, 2006; Chinchilla et al., 2007). Recognition of flg22 and Elf18 leads to an increase in cytosolic Ca^2+^ and it has been show that early signaling is BAK1-dependent and involves calcium associated plasma membrane anion channel opening (Jeworutzki et al., 2010). Subsequently, a MAP kinase cascade involving MPK3, MPK4, MPK6 and MPK11, and other genes such as Ca^2+^-dependent proteinase kinases are activated to establish PTI (Zipfel et al., 2004, 2006; Chinchilla et al., 2007; Bethke et al., 2012).

Pathogenic bacteria secrete, amongst others, type III effectors into plant cells to supress PTI and this mechanism has been well studied in the plant pathogen *P. syringae* (reviewed by Block and Alfano, 2011; Deslandes and Rivas, 2012). These bacterial effectors target a variety of plant genes and metabolites including plasma membrane components like RPM1-interacting protein 4 (RIN4) in *A. thaliana* (Day et al., 2006). Similarly, host nuclear components are, for example, perturbed by effectors such as PopP2 as well as by transcription-activator like (TAL) type III effectors from *Xanthomonas* that directly bind to plant DNA and thereby activate gene expression changes that promote virulence and pathogen colonization (Boch et al., 2009; Deslandes and Rivas, 2012; Coll and Valls, 2013). Other examples include the effector HopAl1 that is widely conserved in bacterial plant pathogens and interferes with the MAPK signaling genes MPK3 and MPK6 to supress PTI (Zhang et al., 2007). Furthermore, chloroplast components are also modified by bacterial effectors such as HopI1 that causes remodeling of the chloroplast thylakoid structure and interferes with SA accumulation (Jelenska et al., 2007). As mentioned above, plants have a variety of *R* genes, the products of which, directly or indirectly, recognize some of these bacterial effectors to elicit ETI (reviewed by Block and Alfano, 2011; Deslandes and Rivas, 2012).

### Oomycete-derived elicitors

Oomycetes are taxonomically and structurally distinct from both plants and fungi. Several oomycetes are plant pathogenic and include those from the genus *Phytophthora* that are responsible for substantial yield losses in crops. Oomycete cell walls consist of cellulose, glycan, and hydroxyproline-rich proteins and several oomycete elicitors have been described (Table 1). For example, necrosis and ethylene-inducing peptide 1 (Nep1)-like proteins (NLP) are recognized in dicots and it has been shown that these proteins trigger a variety of defense responses in *A. thaliana* (Qutob et al., 2006). Similarly, *P. infestans* INF1 elicitin causes an HR response in *Nicotiana benthamiana* (Kamoun et al., 1998) that is dependent on the receptor-like kinase SERK3/BAK1 which, as a central regulator of innate immunity in plants, is required for multiple resistance responses, including those mediated through FLS2 (Heese et al., 2007). Other PTI eliciting molecules from *Phytophthora* include GP42, a member of the transglutaminase family, and for which the active peptide has been described as Pep-13 (Nürnberger et al., 1994; Brunner et al., 2002), as well as the cellulose binding elicitor lectin (CBEL) that is associated with adhesion to the plant cell (Gaulin et al., 2006; reviewed in Hein et al., 2009).

To suppress PTI during infection, *Phytophthora*, like other plant pathogens, secretes extracellular and intracellular effectors into plants. Some extracellular effectors encode protease or glucanase inhibitors to prevent, respectively, host protease or glucanase activity in the apoplast (reviewed in Hein et al., 2009; Schornack et al., 2009). Some intracellular effectors contain the canonical RXLR motif and contain an N-terminal signal peptide and a C-terminal effector activity site (Birch et al., 2009).

The modes of action of RXLR effectors in promoting virulence are diverse. For example, it has recently been shown that the *P. infestans* RXLR effector PexRD2 interacts with the kinase domain of the host MAPKKKε to perturb PTI signaling pathways and to yield ETS responses (King et al., 2014). The RXLR effector PITG_03192, on the other hand, targets two membrane-associated NAC transcription factors that rapidly accumulate following PTI elicitation (McLellan et al., 2013). The effector prevents the release of these NAC transcription factors from the endoplasmic reticulum and subsequent accumulation in the plant nucleus that is typically observed as part of a PTI response. In contrast, the *P. infestans* RXLR effector Avrblb2 prevents secretion of an immune-associated protease (Bozkurt et al., 2011), whereas two *P. sojae* RXLRs have been shown to act as silencing suppressors (Qiao et al., 2013). One of the best-characterized intracellular RXLR effectors is Avr3a from *P*. *infestans*. Avr3a interacts with and stabilizes the potato E3 ubiquitin ligase CMPG1 and thus perturbs cell death responses triggered by INF1 (Bos et al., 2010) and a range of other pathogen elicitors (Gilroy et al., 2011). Avr3a exists in two forms that both suppress INF1 responses but differ in two amino acids that determine recognition by the potato R gene R3 that subsequently triggers ETI (Armstrong et al., 2005). Finally, several RXLRs from *P. infestans* act redundantly to suppress flg22-mediated signal transduction and early transcriptional changes (Zheng et al., 2014).

### Fungal-derived elicitors

As with plant and oomycete cell walls, break-down products from fungal cell walls, which contain chitin, mannoproteins, and β-glucans, can elicit a range of defense responses as signals of potential colonization (Table 1). Yeast extracts, for example, have widely been used to study defense responses in plants (e.g., Hahn and Albersheim, 1978; Reglinski et al., 1994b, 1995; Suzuki et al., 2005; Khokon et al., 2010). Ergosterol, a fungal cell membrane component, induces defense responses in tobacco, and *Cladosporium fulvum* host and non-host plant necrosis inducer 1 (CfHNNI1), which shows high homologies to genes encoding bZIP transcription factors, has been shown to induce resistance in tomato and tobacco (Takken et al., 2000; Xu et al., 2012). Similarly, a proteinaceous elicitor called SCLEROTINIA CULTURE FILTRATE ELICITOR1 (SCFE1) has recently been identified from the necrotrophic fungal pathogen *Sclerotinia sclerotiorum* that induces BAK1-dependent PTI responses upon recognition by the *A. thaliana RECEPTOR-LIKE PROTEIN30 (RLP30)* (Zhang et al., 2013).

Two of the best studied fungal-derived elicitors are chitin and chitosan, a deacetylated derivative of chitin. Both have been well described as active components that increase resistance to bacterial and fungal pathogens in several plant species including crop plants (El Ghaouth et al., 1994; Copping and Duke, 2007; Kishimoto et al., 2010; El Hadrami et al., 2012; Kombrink et al., 2011). Chitin is detected in plants by a chitin elicitor receptor kinase (CERK1) which is also known as LysMRLK1 (Wan et al., 2008; Kombrink et al., 2011). In *A. thaliana*, chitin-induced dimerization of AtCERK1 has shown to be necessary for activation of PTI (Liu et al., 2012). In rice, OsCERK1 forms a complex with chitin elicitor binding protein (CEBiP) upon chitin perception and both proteins are critical for chitin-induced signaling (Shimizu et al., 2010). A homolog of OsCEBiP has been identified in barley and HvCEBiP has also been shown to play a role in responses to *Magnaporthe oryzae* (Tanaka et al., 2010). In contrast to rice, the homolog in *A. thaliana*, AtCEBiP, binds chitin but does not seem to be required for chitin-induced signaling (Shinya et al., 2012). Chitin-induced PTI is JA-, SA-, and ET-independent but a RING zinc-finger like protein (ATL9) has shown to be induced upon chitin treatment (Berrocal-Lobo et al., 2010).

To suppress these responses, the fungal pathogen *C. fulvum* has developed two distinct effectors that suppress chitin-induced PTI leading to ETS *in planta* (de Jonge and Thomma, 2009; de Jonge et al., 2010; Kombrink et al., 2011). The fungal chitin-binding protein Avr4 specifically binds chitin in fungal cell walls and thereby prevents the chitin from degradation by plant chitinases (van den Burg et al., 2006; Wan et al., 2008). Furthermore, the extracellular protein 6 (Ecp6), an effector protein with 3 LysM domains, binds chitin competitively to prevent recognition of chitin by CEBiP (de Jonge and Thomma, 2009; de Jonge et al., 2010). Homologs of Avr4 have been identified in fungi belonging to the class of *Dothideomycetes* and Ecp6-like genes are widespread within the fungal kingdom (Kombrink et al., 2011). The plant receptor Cf4 is a receptor-like protein (RLP) without kinase activity that recognizes Avr4 (Thomas et al., 1997) and it has recently been shown that SOBIR1, a receptor-like kinase (RLK) from tomato interacts with Cf4 and might be required for Cf4-mediated resistance (Liebrand et al., 2013).

Compared with bacterial and oomycete effectors, the biological function and the targets of fungal effectors remain more elusive (Rafiqi et al., 2012; Liu et al., 2013). This has partly been attributed to the fact that fungal effectors do not seem to have canonical amino acid domains that enable a rapid candidate effector discovery (Rafiqi et al., 2012). Recently, Doehlemann and Hemetsberger (2013) reviewed the current knowledge of effectors from filamentous plant pathogens and compiled a list of known apoplastic effectors and their function. Most fungal effectors are secreted through the fungal endoplasmic reticulum (ER) secretory pathway but the way by which cytoplasmic effector proteins enter the host cells remains unknown (Rafiqi et al., 2012). In the genome of *Blumeria graminis*, 491 potential effector proteins have been identified but their biological function remains unknown (Pedersen et al., 2012). Similarly, in *M. oryzae* 15 candidate effector proteins have been identified so far (Liu et al., 2013). An effector protein from *Ustilago maydis* has been identified as a chorismate mutase, Cmu1, which is required for full virulence. Cmu1 functions by diverting metabolic precursors of the shikimate pathway toward production of aromatic amino acids, and away from the production of SA (Djamei and Kahmann, 2012).

## Plant genes and pathways involved in elicitor recognition

### Genes up-regulated due to elicitor treatments

In the elicitor research field, the response of plant genes to elicitor treatment is of great interest and several gene expression studies have been conducted. More recently, several microarray studies have been performed in different plant species to gain greater knowledge of the diversity of genes responsive to elicitors (e.g., Medeiros et al., 2009; Kano et al., 2011; Povero et al., 2011; Amelot et al., 2012), albeit knowledge on plant gene expression in response to elicitors has mainly been focused on *A. thaliana*. As mentioned previously, the presence of the cognate receptors can determine responsiveness to elicitors (Lacombe et al., 2010) and it is thus essential to investigate elicitor effects in diverse crop plants (Nguyen et al., 2010).

As part of this review, we aim to provide an overview of the current knowledge of differentially expressed plant genes following elicitor treatments and identify typically affected plant processes. This will facilitate identification of responses to elicitor application such as plant growth or nutrient metabolisms that are not directly linked to defense but impact on agriculture. For this we have combined over 50 publications to create a list of plant genes that are differentially expressed following the recognition of elicitors (Table S1). Reciprocal BLAST (Basic Local Alignment Search Tool; Altschul et al., 1990) has been used to identify the *A. thaliana* homologs when the original experiment was performed in a different plant species (>70% identity of nucleotide sequences, *E*-value < 0.0001). In addition to the publications, PathoPlant®, a database featuring compiled expression data and components of signal transduction pathways related to plant pathogenesis, has been used (Bülow et al., 2004, 2007). This database enables querying differential plant gene expression following diverse pathogen stimuli which, for this study, include *Botrytis cinerea*, chitin, *Erysiphe orontii*, *Phytophthora infestans*, *Pseudomonas syringae* pv. Maculicola, and *Pseudomonas syringae* pv. Phaseolicola.

A total of 1592 plant genes that were activated by the recognition of elicitors have been identified (Table S1). Ontological analysis was performed using agriGo (Du et al., 2010). In this analysis, genes of interest are grouped by gene ontology (GO) terms describing biological processes, molecular functions and cellular components (Ashburner et al., 2000). The list of genes of interest is compared to a defined background gene list which, in this study, included the whole genome of *A. thaliana* as provided by The Arabidopsis Information Resource (TAIR) (Lamesch et al., 2011) to identify GO terms that are significantly over-represented. A total of 762 shared GO terms were identified, comprising 474 biological processes, 206 molecular functions and 82 cellular components (Table S2).

**Figure 2:**
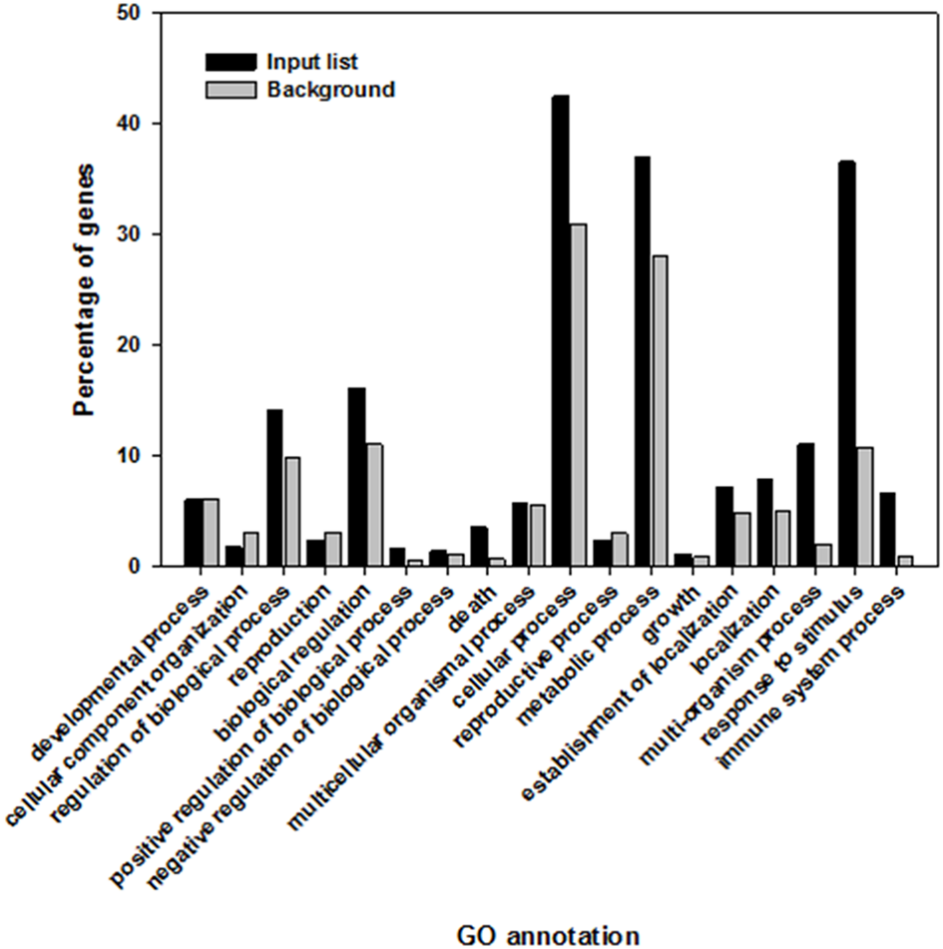
**Highly significant shared biological processes within *Arabidopsis thaliana* genes that are induced and overrepresented following the recognition of elicitor compounds (black) in comparison to the whole genome of *A. thaliana* (gray)**.

The three most over-represented biological processes were “response to stimulus” (GO:0050896), “multi-organism process” (GO:0051704), and “immune system process” (GO:0002376). The GO term “response to stimulus” contained 36.5% of the elicitor responsive genes in comparison to 10.7% representation in the whole *A. thaliana* genome. The GO term “multi-organism process” contained 11.1% of the genes in the target gene list, compared to 2.1% of the whole genome, and 6.6% instead of 1% were classed in the GO term “immune system process” (Figure 2).

**Figure 3:**
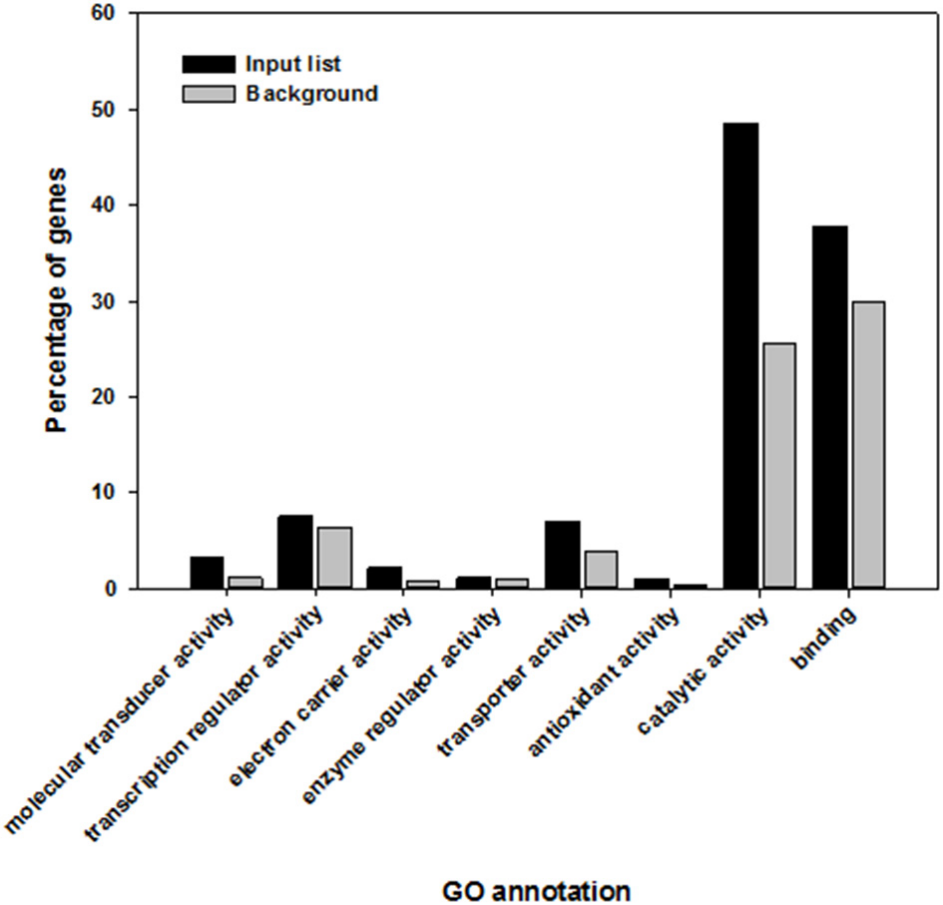
**Highly significant shared molecular functions within *Arabidopsis thaliana* genes that are induced and overrepresented following the recognition of elicitor compounds (black) in comparison to the whole genome of *A. thaliana* (gray)**.

Three of the over-represented molecular functions were “catalytic activity” (GO:0003824), “binding” (GO:0005488), and “molecular transducer activity” (GO:0060089). The GO term “catalytic activity” featured in 48.5% of the elicitor responsive genes in comparison to 25.5% of the whole *A. thaliana* genome. The GO term “binding” contained 37.8% of the genes in the target gene list in comparison to 29.8% of the whole genome and 3.1% instead of 1.1% were classed in the GO term “molecular transducer activity” (Figure 3).

The relationships of all over-represented genes in the classification “biological processes” are shown in Figure S1. These include metabolic processes such as amine-, phosphate-, and phytoalexin metabolism; immune system processes and cell death, including regulation of defense response; plant-type hypersensitive response and apoptosis; response to stimuli including JA and SA; systemic acquired resistance and defense responses to fungi and bacteria (Figure S1).

The relationship of all genes over-represented in the GO term “molecular functions” are shown in Figure S2. These functions contain catalytic activity including oxidoreductase, lyase, and kinase activities; and binding activities including ATP and sugar binding (Figure S2). The relationships of all over-represented genes in the classification “cellular components” are shown in Figure S3. All cell parts are involved but the involvements of cell wall and plasma membranes are highly significant (Figure S3).

### Targets of pathogen effectors

As noted above, pathogens produce effector molecules to interfere with plant defense responses. An analysis of plant-pathogen protein-protein interactions using *A. thaliana* and two pathogens, *P. syringae* and the obligate biotrophic oomycete *Hyaloperonospora arabidopsidis* revealed 137 *A. thaliana* proteins that were potentially targeted by pathogen effectors (Mukhtar et al., 2011). A recent review on bacterial effectors listed an additional 22 plant proteins targeted by several bacterial effectors (Deslandes and Rivas, 2012). In the analysis here, genes encoding these 159 proteins were used to search for overlap with the plant genes differentially induced upon elicitor recognition. A total of 23 genes were identified that are both induced by elicitors and targeted by pathogen effectors (Table S3). These comprise receptors such as FLS2 and EFR, genes involved in MAPK cascades like RIPK, MPK3, MPK4, and MPK6, R protein-guarded host proteins such as RIN4 and genes involved in L-phenylalanine biosynthetic process like ADT4 and ADT5 (Table S3). These overlapping genes are grouped into several biological processes, molecular functions and cell parts and their involvements are highlighted with stars in Figures S1–S3. This analysis highlights the complexity of priming and plant immune responses, and the sophisticated interactions with pathogen effectors. It shows that the plant response to elicitor compounds does not only involve genes that are annotated as defense-related but that other metabolic pathways are also involved. We know that elicitor treatments result in positive and negative trade-offs (Walters and Heil, 2007) and therefore expression profiling of some of these genes should result in a better understanding of these responses and how they might be exploited.

## “Non-defense” effects of elicitors

As mentioned above, for successful use of elicitors in agriculture it is important to understand their effects not only on plant defense but also on other aspects of plant development and environmental responses. The activation of defense pathways as part of PTI can be very costly to plants but should be less than the potential loss caused by disease if no defense was mounted. However, in the context of crop protection, such costs are unlikely to be acceptable in the absence of known pathogen challenges of a high order. Crop protectants are preferred that enhance the efficacy of PTI assisting a quicker and more effective response when an actual pathogen challenge occurs and therefore is more efficient in its use of resources. This can be achieved through priming if priming is either not costly to the plant or its costs are mitigated by other beneficial means.

Some of the known priming genes are generally regulatory but not necessarily restricted to defense pathways. They regulate signal transduction events, particularly those identified in stress responses or in the GO terms “response to stimulus,” “multi-organism processes,” and “immune system processes,” i.e., the genes disproportionately up-regulated by elicitors highlighted above (see Figures S1, S2). Thus, many non-defense mechanism processes will be affected in their expression by elicitors. These might result in additional costs above those incurred by defense gene expression, but they may also have benefits. An example of a non-defense effect of an elicitor is reduced water use of pepper plants upon treatment with chitosan (Bittelli et al., 2001). Chitosan was also found to affect the net photosynthetic rate of soybean and maize after application (Khan et al., 2002). More general effects on yield, not directly attributable to disease control, were also found from applications of *B. subtilis* culture filtrates (Dehne et al., 1984; Steiner et al., 1988). Similar effects were recorded for some treatments of yeast cell wall-derived extracts (Reglinski et al., 1994a).

Indeed, on a molecular level, there is evidence of cross talk between the MAPKs involved in PTI and abiotic stress responses. For example, the transcription of MEKK1 is induced by diverse stresses including cold, salt, drought and wounding (Mizoguchi et al., 1998). Conversely, the activation of EDS1/PAD4-dependent signaling during ETI responses can rapidly antagonize ABA signal transduction at the level of Ca^2+^ signaling (Kim et al., 2011). The overexpression of the gene ACTIVATED DISEASE RESISTANCE1 (ADS1) in *A. thaliana*, a member of the nucleotide-binding (NB) and leucine-rich repeat (LRR) containing NB-LRR genes, confers both disease resistance (Grant et al., 2003) and drought tolerance, requiring SA, EDS1 and ABA-INSENSITIVE1 (ABI1) (Chini et al., 2004).

Much depends on the basis of determination of costs as we tend to calculate these from an end-user yield perspective. We should also recognize that each of these pathways, whether defense-related or not, is in a complex expression and metabolic network of cross-talk and feedback mechanisms and thus affected by many environmental factors. Those that can be manipulated beneficially and perhaps synergistically fall in the category of nutrition (Walters and Bingham, 2007). Primed plants showed considerably higher fitness than non-primed plants when they were challenged by pathogens without major trade-off effects on growth and seed set (Conrath et al., 2006; van Hulten et al., 2006). Correlation of priming benefits with gene expression profiles may lead to very practical means for developing elicitor-based crop protectants that either off-set any direct costs, increase some aspect of resource use efficiency or specifically enhance other processes beneficial to yield or quality.

### Conflict of interest statement

The authors declare that the research was conducted in the absence of any commercial or financial relationships that could be construed as a potential conflict of interest.

## References

[B1] Altschul S. F. Gish W. Miller W. Myers E. W. Lipman D. J. (1990). Basic local alignment search tool. J. Mol. Biol. 215, 403–410. 10.1016/S0022-2836(05)80360-2 2231712

[B2] Amelot N. de Borne F. D. Clemente H. S. Mazars C. Grima-Pettenati J. Briere C. (2012). Transcriptome analysis of tobacco BY-2 cells elicited by cryptogein reveals new potential actors of calcium-dependent and calcium-independent plant defense pathways. Cell Calcium 51, 117–130. 10.1016/j.ceca.2011.11.010 22177386

[B3] Armstrong M. R. Whisson S. C. Pritchard L. Bos J. I. Venter E. Avrova A. O.. (2005). An ancestral oomycete locus contains late blight avirulence gene *Avr3a*, encoding a protein that is recognized in the host cytoplasm. Proc. Natl. Acad. Sci. U.S.A. 102, 7766–7771. 10.1073/pnas.0500113102 15894622 PMC1140420

[B4] Ashburner M. Ball C. A. Blake J. A. Botstein D. Butler H. Cherry J. M.. (2000). Gene Ontology: tool for the unification of biology. Nat. Genet. 25, 25–29. 10.1038/75556 10802651 PMC3037419

[B5] Aziz A. Gauthier A. Bézier A. Poinssot B. Joubert J. M. Pugin A.. (2007). Elicitor and resistance-inducing activities of β-1,4 cellodextrins in grapevine, comparison with β-1,3 glucans and α-1,4 oligogalacturonides. J. Exp. Bot. 58, 1463–1472. 10.1093/jxb/erm008 17322548

[B6] Beckers G. J. Jaskiewicz M. Liu Y. Underwood W. R. He S. Y. Zhang S.. (2009). Mitogen-activated protein kinases 3 and 6 are required for full priming of stress responses in *Arabidopsis thaliana*. Plant Cell 21, 944–953. 10.1105/tpc.108.062158 19318610 PMC2671697

[B7] Berrocal-Lobo M. Stone S. Yang X. Antico J. Callis J. Ramonell K. M.. (2010). ATL9, a RING zinc finger protein with E3 ubiquitin ligase activity implicated in chitin- and NADPH oxidase-mediated defense responses. PLoS ONE 5:e14426. 10.1371/journal.pone.0014426 21203445 PMC3009710

[B8] Bethke G. Pecher P. Eschen-Lippold L. Tsuda K. Katagiri F. Glazebrook J.. (2012). Activation of the *Arabidopsis thaliana* mitogen-activated protein kinase MPK11 by the flagellin-derived elicitor peptide, flg22. Mol. Plant Microbe Interact. 25, 471–480. 10.1094/MPMI-11-11-0281 22204645

[B9] Birch P. R. Armstrong M. Bos J. Boevink P. Gilroy E. M. Taylor R. M.. (2009). Towards understanding the virulence functions of RXLR effectors of the oomycete plant pathogen *Phytophthora infestans*. J. Exp. Bot. 60, 1133–1140. 10.1093/jxb/ern353 19204033

[B10] Bishop G. J. Koncz C. (2002). Brassinosteroids and plant steroid hormone signaling. Plant Cell 14, S97–S110. 10.1105/tpc.001461 12045272 PMC151250

[B11] Bittel P. Robatzek S. (2007). Microbe-associated molecular patterns (MAMPs) probe plant immunity. Curr. Opin. Plant Biol. 10, 335–341. 10.1016/j.pbi.2007.04.021 17652011

[B12] Bittelli M. Flury M. Campbell G. S. Nichols E. J. (2001). Reduction of transpiration through foliar application of chitosan. Agr. Forest Meteorol. 107, 167–175 10.1016/S0168-1923(00)00242-2

[B13] Block A. Alfano J. R. (2011). Plant targets for *Pseudomonas syringae* type III effectors: virulence targets or guarded decoys? Curr. Opin. Plant Biol. 14, 39–46. 10.1016/j.mib.2010.12.011 21227738 PMC3040236

[B14] Boch J. Scholze H. Schornack S. Landgraf A. Hahn S. Kay S.. (2009). Breaking the code of DNA binding specificity of TAL-type III effectors. Science 326, 1509–1512. 10.1126/science.1178811 19933107

[B15] Bonnet P. Bourdon E. Ponchet M. Blein J. P. Ricci P. (1996). Acquired resistance triggered by elicitins in tobacco and other plants. Eur. J. Plant Pathol. 102, 181–192 10.1007/BF01877105

[B16] Bos J. I. B. Armstrong M. R. Gilroy E. M. Boevink P. C. Hein I. Taylor R. M.. (2010). *Phytophthora infestans* effector AVR3a is essential for virulence and manipulates plant immunity by stabilizing host E3 ligase CMPG1. Proc. Natl. Acad. Sci. U.S.A. 107, 9909–9914. 10.1073/pnas.0914408107 20457921 PMC2906857

[B17] Bourque S. Dutartre A. Hammoudi V. Blanc S. Dahan J. Jeandroz S.. (2011). Type-2 histone deacetylases as new regulators of elicitor-induced cell death in plants. New Phytol. 192, 127–139. 10.1111/j.1469-8137.2011.03788.x 21651563

[B18] Bozkurt T. O. Schornack S. Win J. Shindo T. Ilyas M. Oliva R.. (2011). *Phytophthora infestans* effector Avrblb2 prevents secretion of a plant immune protease at the haustorial interface. Proc. Natl. Acad. Sci. U.S.A. 108, 20832–20837. 10.1073/pnas.1112708109 22143776 PMC3251060

[B19] Brunner F. Rosahl S. Lee J. Rudd J. J. Geiler C. Kauppinen S.. (2002). Pep-13, a plant defense-inducing pathogen-associated pattern from *Phytophthora* transglutaminases. EMBO J. 21, 6681–6688. 10.1093/emboj/cdf667 12485989 PMC139088

[B20] Brutus A. Sicilia F. Macone A. Cervone F. De Lorenzo G. (2010). A domain swap approach reveals a role of the plant wall-associated kinase 1 (WAK1) as a receptor of oligogalacturonides. Proc. Natl. Acad. Sci. U.S.A. 107, 9452–9457. 10.1073/pnas.1000675107 20439716 PMC2889104

[B21] Bülow L. Schindler M. Choi C. Hehl R. (2004). PathoPlant®: a database on plant-pathogen interactions. In Silico Biol. 4, 529–536. 15752070

[B22] Bülow L. Schindler M. Hehl R. (2007). PathoPlant®: a platform for microarray expression data to analyze co-regulated genes involved in plant defense responses. Nucleic Acids Res. 35, D841–D845. 10.1093/nar/gkl835 17099232 PMC1669748

[B23] Chen M. Zeng H. Qiu D. Guo L. Yang X. Shi H.. (2012). Purification and characterization of a novel hypersensitive response-inducing elicitor from *Magnaporthe oryzae* that triggers defense response in rice. PLoS ONE 7:e37654. 10.1371/journal.pone.0037654 22624059 PMC3356297

[B24] Chen Z. Zheng Z. Huang J. Lai Z. Fan B. (2009). Biosynthesis of salicylic acid in plants. Plant Signal. Behav. 4, 493–496. 10.4161/psb.4.6.8392 19816125 PMC2688294

[B25] Chinchilla D. Zipfel C. Robatzek S. Kemmerling B. Nürnberger T. Jones J. D. G.. (2007). A flagellin-induced complex of the receptor FLS2 and BAK1 initiates plant defence. Nature 448, 497–500. 10.1038/nature05999 17625569

[B26] Chini A. Grant J. J. Seki M. Shinozaki K. Loake G. J. (2004). Drought tolerance established by enhanced expression of the CC-NBS-LRR gene, ADR1, requires salicylic acid, EDS1 and ABI1. Plant J. 385, 810–822. 10.1111/j.1365-313X.2004.02086.x 15144382

[B27] Cluzet S. Torregrosa C. Jacquet C. Lafitte C. Fournier J. Mercier L. (2004). Gene expression profiling and protection of *Medicago truncatula* against a fungal infection in response to an elicitor from green algae *Ulva* spp. Plant Cell Environ. 27, 917–928 10.1111/j.1365-3040.2004.01197.x

[B28] Coll N. S. Valls M. (2013). Current knowledge on the *Ralstonia solanacearum* type III secretion system. Microb. Biotechnol. 6, 614–620. 10.1111/1751-7915.12056 23617636 PMC3815929

[B29] Conrath U. (2011). Molecular aspects of defence priming. Trends Plant Sci. 16, 524–531. 10.1016/j.tplants.2011.06.004 21782492

[B30] Conrath U. Beckers G. J. Flors V. Garcia-Agustin P. Jakab G. Mauch F.. (2006). Priming: getting ready for battle. Mol. Plant Microbe Interact. 19, 1062–1071. 10.1094/MPMI-19-1062 17022170

[B31] Copping L. G. Duke S. O. (2007). Natural products that have been used commercially as crop protection agents. Pest Manag. Sci. 63, 524–554. 10.1002/ps.1378 17487882

[B32] Craigie J. S. (2011). Seaweed extract stimuli in plant science and agriculture. J. Appl. Phycol. 23, 371–393 10.1007/s10811-010-9560-4

[B33] Daayf F. Ongena M. Boulanger R. El Hadrami I. Bélanger R. R. (2000). Induction of phenolic compounds in two cultivars of cucumber by treatment of healthy and powdery mildew-infected plants with extracts of *Reynoutria sachalinensis*. J. Chem. Ecol. 26, 1579–1593 10.1023/A:1005578510954

[B34] Daayf F. Schmitt A. Belanger R. R. (1997). Evidence of phytoalexins in cucumber leaves infected with powdery mildew following treatment with leaf extracts of *Reynoutria sachalinensis*. Plant Physiol. 113, 719–727. 12223638 10.1104/pp.113.3.719 PMC158189

[B35] Danna C. H. Millet Y. A. Koller T. Han S. W. Bent A. F. Ronald P. C.. (2011). The *Arabidopsis* flagellin receptor FLS2 mediates the perception of *Xanthomonas* Ax21 secreted peptides. Proc. Natl. Acad. Sci. U.S.A. 108, 9286–9291. 10.1073/pnas.1106366108 21576467 PMC3107323

[B36] Day B. Dahlbeck D. Staskawicz B. J. (2006). NDR1 interaction with RIN4 mediates the differential activation of multiple disease resistance pathways in *Arabidopsis*. Plant Cell 18, 2782–2791. 10.1105/tpc.106.044693 17012600 PMC1626609

[B37] Dehne H.-W. Stenzel K. Schönbeck F. (1984). Zur Wirksamkeit induzierter Resistenz unter praktischen Anbaubedingungen III. Reproduktion echter Mehltaupilze auf induziert resistenten Pflanzen. Z. Pflanzenk. Pflanzen. 91, 258–265.

[B38] de Jonge R. Peter van Esse H. Kombrink A. Shinya T. Desaki Y. Bours R.. (2010). Conserved fungal LysM effector Ecp6 prevents chitin-triggered immunity in plants. Science 329, 953–955. 10.1126/science.1190859 20724636

[B39] de Jonge R. Thomma B. P. (2009). Fungal LysM effectors: extinguishers of host immunity? Trends Microbiol. 17, 151–157. 10.1016/j.tim.2009.01.002 19299132

[B40] de Oliveira A. L. Gallo M. Pazzagli L. Benedetti C. E. Cappugi G. Scala A.. (2011). The structure of the elicitor cerato-platanin (CP), the first member of the CP fungal protein family, reveals a double ψβ-barrel fold and carbohydrate binding. J. Biol. Chem. 286, 17560–17568. 10.1074/jbc.M111.223644 21454637 PMC3093830

[B41] Desaki Y. Miya A. Venkatesh B. Tsuyumu S. Yamane H. Kaku H.. (2006). Bacterial lipopolysaccharides induce defense responses associated with programmed cell death in rice cells. Plant Cell Physiol. 47, 1530–1540. 10.1093/pcp/pcl019 17018557

[B42] Deslandes L. Rivas S. (2012). Catch me if you can: bacterial effectors and plant targets. Trends Plant Sci. 17, 644–655. 10.1016/j.tplants.2012.06.011 22796464

[B43] De Vleesschauwer D. Höfte M. (2009). Rhizobacteria-induced systemic resistance. Adv. Bot. Res. 51, 223–281 10.1016/S0065-2296(09)51006-3

[B44] Djamei A. Kahmann R. (2012). *Ustilago maydis*: dissecting the molecular interface between pathogen and plant. PLoS Pathog. 8:e1002955. 10.1371/journal.ppat.1002955 23133380 PMC3486881

[B45] Djonoviç S. Vargas W. A. Kolomiets M. V. Horndeski M. Wiest A. Kenerley C. M. (2007). A proteinaceous elicitor Sm1 from the beneficial fungus *Trichoderma virens* is required for induced systemic resistance in maize. Plant Physiol. 145, 875–889. 10.1104/pp.107.103689 17885089 PMC2048795

[B46] Doehlemann G. Hemetsberger C. (2013). Apoplastic immunity and its suppression by filamentous plant pathogens. New Phytol. 198, 1001–1016. 10.1111/nph.12277 23594392

[B47] Dow M. Newman M. A. von Roepenack E. (2000). The induction and modulation of plant defense responses by bacterial lipopolysaccharides. Annu. Rev. Phytopathol. 38, 241–261. 10.1146/annurev.phyto.38.1.241 11701843

[B48] Du Z. Zhou X. Ling Y. Zhang Z. Su Z. (2010). agriGO: a GO analysis toolkit for the agricultural community. Nucleic Acids Res. 38, W64–W70. 10.1093/nar/gkq310 20435677 PMC2896167

[B49] El Ghaouth A. Arul J. Grenier J. Benhamou N. Asselin A. Bélanger R. (1994). Effect of chitosan on cucumber plants: suppression of *Pythium aphanidermatum* and induction of defense reactions. Phytopathology 84, 313–320 10.1094/Phyto-84-313

[B50] El Hadrami A. Adam L. R. El Hadrami I. Daayf F. (2012). Chitosan in plant protection. Mar. Drugs 8, 968–987. 10.3390/md8040968 20479963 PMC2866471

[B51] Erbs G. Newman M. A. (2012). The role of lipopolysaccharide and peptidoglycan, two glycosylated bacterial microbe-associated molecular patterns (MAMPs), in plant innate immunity. Mol. Plant Pathol. 13, 95–104. 10.1111/j.1364-3703.2011.00730.x 21726397 PMC6638628

[B52] Eshraghi L. Anderson J. Aryamanesh N. Shearer B. McComb J. Hardy G. E. S. (2011). Phosphite primed defence responses and enhanced expression of defence genes in *Arabidopsis thaliana* infected with *Phytophthora cinnamomi*. Plant Pathol. 60, 1086–1095 10.1111/j.1365-3059.2011.02471.x

[B53] Eulgem T. (2005). Regulation of the *Arabidopsis* defense transcriptome. Trends Plant Sci. 10, 71–78. 10.1016/j.tplants.2004.12.006 15708344

[B54] Falcón-Rodríguez A. B. Wegria G. Cabrera J.-C. (2012). Exploiting plant innate immunity to protect crops against biotic stress: chitosaccharides as natural and suitable candidates for this purpose, in New Perspectives in Plant Protection, ed Bandani A. R. (Rijeka: In Tech Croatia), 139–166.

[B55] Felix G. Boller T. (2003). Molecular sensing of bacteria in plants. The highly conserved RNA-binding motif RNP-1 of bacterial cold shock proteins is recognized as an elicitor signal in tobacco. J. Biol. Chem. 278, 6201–6208. 10.1074/jbc.M209880200 12471032

[B56] Ferrari S. Savatin D. V. Sicilia F. Gramegna G. Cervone F. De Lorenzo G. (2013). Oligogalacturonides: plant damage-associated molecular patterns and regulators of growth and development. Front. Plant Sci. 4:49. 10.3389/fpls.2013.00049 23493833 PMC3595604

[B57] Flors V. Ton J. Jakab G. Mauch-Mani B. (2005). Abscisic acid and callose: team players in defence against pathogens? J. Phytopathol. 153, 377–383 10.1111/j.1439-0434.2005.00987.x

[B58] Fu Y. Yin H. Wang W. Wang M. Zhang H. Zhao X. (2011). β-1,3-glucan with different degree of polymerization induced different defense responses in tobacco. Carbohyd. Polym. 86, 774–782 10.1016/j.carbpol.2011.05.022

[B59] Galletti R. Ferrari S. De Lorenzo G. (2011). Arabidopsis MPK3 and MPK6 play different roles in basal and oligogalacturonide- or flagellin-induced resistance against *Botrytis cinerea*. Plant Physiol. 157, 804–814. 10.1104/pp.111.174003 21803860 PMC3192574

[B60] Garcion C. Lamotte O. Métraux J.-P. (2007). Mechanisms of defense to pathogens: biochemistry and physiology, in Induced Resistance for Plant Defence, eds Walters D. R. Newton A. C. Lyon G. D. (Oxford: Blackwell Publishing), 109–132 10.1002/9780470995983.ch6

[B61] Gaulin E. Dramé N. Lafitte C. Torto-Alalibo T. Martinez Y. Ameline-Torregrosa C.. (2006). Cellulose binding domains of a *Phytophthora* cell wall protein are novel pathogen-associated molecular patterns. Plant Cell 18, 1766–1777. 10.1105/tpc.105.038687 16766692 PMC1488925

[B62] Gerber I. Zeidler D. Durner J. Dubery I. (2004). Early perception responses of *Nicotiana tabacum* cells in response to lipopolysaccharides from *Burkholderia cepacia*. Planta 218, 647–657. 10.1007/s00425-003-1142-0 14605884

[B63] Gilroy E. M. Taylor R. M. Hein I. Boevink P. Sadanandom A. Birch P. R. J. (2011). CMPG1-dependent cell death follows perception of diverse pathogen elicitors at the host plasma membrane and is suppressed by *Phytophthora infestans* RXLR effector AVR3a. New Phytol. 190, 653–666. 10.1111/j.1469-8137.2011.03643.x 21348873

[B64] Gomez-Gomez L. Boller T. (2002). Flagellin perception: a paradigm for innate immunity. Trends Plant Sci. 7, 251–256. 10.1016/S1360-1385(02)02261-6 12049921

[B65] Goupil P. Benouaret R. Charrier O. Ter Halle A. Richard C. Eyheraguibel B.. (2012). Grape marc extract acts as elicitor of plant defence responses. Ecotoxicology 21, 1541–1549. 10.1007/s10646-012-0908-1 22547210

[B66] Grant J. J. Chini A. Basu D. Loake G. J. (2003). Targeted activation tagging of the Arabidopsis NBS-LRR gene, ADR1, conveys resistance to virulent pathogens. Mol. Plant Microbe Interact. 16, 669–680. 10.1094/MPMI.2003.16.8.669 12906111

[B67] Guo M. Chen K. Zhang P. (2012). Transcriptome profile analysis of resistance induced by burdock fructooligosaccharide in tobacco. J. Plant Physiol. 169, 1511–1519. 10.1016/j.jplph.2012.06.019 22921678

[B68] Hahn M. G. Albersheim P. (1978). Host-pathogen interactions: XIV. isolation and partial characterization of an elicitor from yeast extract. Plant Physiol. 62, 107–111. 10.1104/pp.62.1.107 16660446 PMC1092066

[B69] Heese A. Hann D. R. Gimenez-Ibanez S. Jones A. M. He K. Li J.. (2007). The receptor-like kinase SERK3/BAK1 is a central regulator of innate immunity in plants. Proc. Natl. Acad. Sci. U.S.A. 104, 12217–12222. 10.1073/pnas.0705306104 17626179 PMC1924592

[B70] Hein I. Gilroy E. M. Armstrong M. R. Birch P. R. J. (2009). The zig-zag-zig in oomycete?plant interactions. Mol. Plant Pathol. 10, 547–562. 10.1111/j.1364-3703.2009.00547.x 19523107 PMC6640229

[B71] Henriquez M. A. Wolski E. A. Molina O. I. Adam L. R. Andreu A. B. Daayf F. (2012). Effects of glucans and eicosapentaenoic acid on differential regulation of phenylpropanoid and mevalonic pathways during potato response to *Phytophthora infestans*. Plant Physiol. Biochem. 60, 119–128. 10.1016/j.plaphy.2012.07.027 22922112

[B72] Henry G. Thonart P. Ongena M. (2012). PAMPs, MAMPs, DAMPs and others: an update on the diversity of plant immunity elicitors. Biotechnol. Agron. Soc. Environ. 16, 257–268.

[B73] Huang C.-J. Tsay J.-F. Chang S.-Y. Yang H.-P. Wu W.-S. Chen C.-Y. (2012). Dimethyl disulfide is an induced systemic resistance elicitor produced by *Bacillus cereus* C1L. Pest Manag. Sci. 68, 1306–1310. 10.1002/ps.3301 22573612

[B74] Huffaker A. Dafoe N. J. Schmelz E. A. (2011). ZmPep1, an ortholog of Arabidopsis elicitor peptide 1, regulates maize innate immunity and enhances disease resistance. Plant Physiol. 155, 1325–1338. 10.1104/pp.110.166710 21205619 PMC3046589

[B75] Igarashi D. Takeda T. Narusaka Y. Totsuka K. (2010). Glutamate fermentation by-product activates plant defence responses and confers resistance against pathogen infection. J. Phytopathol. 158, 668–675 10.1111/j.1439-0434.2010.01678.x

[B76] Jaskiewicz M. Conrath U. Peterhaensel C. (2011). Chromatin modification acts as a memory for systemic acquired resistance in the plant stress response. EMBO Rep. 12, 50–55. 10.1038/embor.2010.186 21132017 PMC3024125

[B77] Jaulneau V. Lafitte C. Corio-Costet M. F. Stadnik M. J. Salamagne S. Briand X. (2011). An *Ulva armoricana* extract protects plants against three powdery mildew pathogens. Eur. J. Plant Pathol. 131, 393–401 10.1007/s10658-011-9816-0

[B78] Jelenska J. Yao N. Vinatzer B. A. Wright C. M. Brodsky J. L. Greenberg J. T. (2007). A J domain virulence effector of *Pseudomonas syringae* remodels host chloroplasts and suppresses defenses. Curr. Biol. 17, 499–508. 10.1016/j.cub.2007.02.028 17350264 PMC1857343

[B79] Jeworutzki E. Roelfsema M. Anschütz U. Krol E. Elzenga J. Felix G.. (2010). Early signaling through the Arabidopsis pattern recognition receptors FLS2 and EFR involves Ca^2+^-associated opening of plasma membrane anion channels. Plant J. 62, 367–378. 10.1111/j.1365-313X.2010.04155.x 20113440

[B80] Jin W. Wu F. Xiao L. Liang G. Zhen Y. Guo Z. (2012). Microarray-based analysis of tomato miRNA regulated by *Botrytis cinerea*. J. Plant Growth Regul. 31, 38–46 10.1007/s00344-011-9217-9

[B81] Jones J. D. Dangl J. L. (2006). The plant immune system. Nature 444, 323–329. 10.1038/nature05286 17108957

[B82] Kamoun S. van West P. Vleeshouwers V. G. A. A. de Groot K. E. Govers F. (1998). Resistance of *Nicotiana benthamiana* to *Phytophthora infestans* is mediated by the recognition of the elicitor protein INF1. Plant Cell 10, 1413–1425. 10.1105/tpc.10.9.1413 9724689 PMC144078

[B83] Kano A. Hosotani K. Gomi K. Yamasaki-Kokudo Y. Shirakawa C. Fukumoto T.. (2011). D-Psicose induces upregulation of defense-related genes and resistance in rice against bacterial blight. J. Plant Physiol. 168, 1852–1857. 10.1016/j.jplph.2011.04.003 21601944

[B85] Kawaguchi Y. Nishiuchi T. Kodama H. Nakano T. Nishimura K. Shimamura K. (2012). Fungal elicitor-induced retardation and its restoration of root growth in tobacco seedlings. Plant Growth Regul. 66, 59–68 10.1007/s10725-011-9629-3

[B86] Kawamura Y. Hase S. Takenaka S. Kanayama Y. Yoshioka H. Kamoun S. (2009). INF1 elicitin activates jasmonic acid- and ethylene-mediated signalling pathways and induces resistance to bacterial wilt disease in tomato. J. Phytopathol. 157, 287–297 10.1111/j.1439-0434.2008.01489.x

[B87] Khan W. M. Prithiviraj B. Smith D. L. (2002). Effect of foliar application of chitin and chitosan oligosaccharides on photosynthesis of maize and soybean. Photosynthetica 40, 621–624 10.1023/A:1024320606812

[B88] Khatib M. Lafitte C. Esquerré-Tugayé M.-T. Bottin A. Rickauer M. (2004). The CBEL elicitor of *Phytophthora parasitica* var. *nicotianae* activates defence in *Arabidopsis thaliana* via three different signalling pathways. New Phytol. 162, 501–510 10.1111/j.1469-8137.2004.01043.x

[B89] Khokon M. Hossain M. A. Munemasa S. Uraji M. Nakamura Y. Mori I. C.. (2010). Yeast elicitor-induced stomatal closure and peroxidase-mediated ROS production in Arabidopsis. Plant Cell Physiol. 51, 1915–1921. 10.1093/pcp/pcq145 20876608

[B90] Kim T. H. Hauser F. Ha T. Xue S. Böhmer M. Nishimura N.. (2011). Chemical genetics reveals negative regulation of abscisic acid signaling by a plant immune response pathway. Curr. Biol. 21, 990–997. 10.1016/j.cub.2011.04.045 21620700 PMC3109272

[B91] King S. R. F. McLellan H. Boevink P. C. Armstrong M. R. Bukharova T. Sukarta O.. (2014). *Phytophthora infestans* RXLR effector PexRD2 interacts with host MAPKKK? to suppress plant immune signaling. Plant Cell 26, 1345–1359. 10.1105/tpc.113.120055 24632534 PMC4001388

[B92] Kishimoto K. Kouzai Y. Kaku H. Shibuya N. Minami E. Nishizawa Y. (2010). Perception of the chitin oligosaccharides contributes to disease resistance to blast fungus *Magnaporthe oryzae* in rice. Plant J. 64, 343–354. 10.1111/j.1365-313X.2010.04328.x 21070413

[B93] Knoth C. Salus M. S. Girke T. Eulgem T. (2009). The synthetic elicitor 3,5-dichloroanthranilic acid induces *NPR1*-dependent and *NPR1*-independent mechanisms of disease resistance in Arabidopsis. Plant Physiol. 150, 333–347. 10.1104/pp.108.133678 19304930 PMC2675713

[B94] Kohler A. Schwindling S. Conrath U. (2002). Benzothiadiazole-induced priming for potentiated responses to pathogen infection, wounding, and infiltration of water into leaves requires the *NPR1/NIM1* gene in Arabidopsis. Plant Physiol. 128, 1046–1056. 10.1104/pp.010744 11891259 PMC152216

[B95] Kombrink A. Sanchez-Vallet A. Thomma B. P. (2011). The role of chitin detection in plant-pathogen interactions. Microb. Infect. 13, 1168–1176. 10.1016/j.micinf.2011.07.010 21856436

[B96] Konstantinidou-Doltsinis S. Markellou E. Kasselaki A. M. Fanouraki M. N. Koumaki C. M. Schmitt A. (2006). Efficacy of Milsana®, a formulated plant extract from *Reynoutria sachalinensis*, against powdery mildew of tomato (*Leveillula taurica*). Biocontrol 51, 375–392 10.1007/s10526-005-5247-1

[B97] Koschmann J. Machens F. Becker M. Niemeyer J. Schulze J. Bülow L.. (2012). Integration of bioinformatics and synthetic promoters leads to the discovery of novel elicitor-responsive cis-regulatory sequences in Arabidopsis. Plant Physiol. 160, 178–191. 10.1104/pp.112.198259 22744985 PMC3440196

[B98] Kulye M. Liu H. Zhang Y. Zeng H. Yang X. Qiu D. (2012). Hrip1, a novel protein elicitor from necrotrophic fungus, *Alternaria tenuissima*, elicits cell death, expression of defence-related genes and systemic acquired resistance in tobacco. Plant Cell Environ. 35, 2104–2120. 10.1111/j.1365-3040.2012.02539.x 22591019

[B99] Kunze G. Zipfel C. Robatzek S. Niehaus K. Boller T. Felix G. (2004). The N terminus of bacterial elongation factor Tu elicits innate immunity in Arabidopsis plants. Plant Cell 16, 3496–3507. 10.1105/tpc.104.026765 15548740 PMC535888

[B100] Lacombe S. Rougon-Cardoso A. Sherwood E. Peeters N. Dahlbeck D. van Esse H. P.. (2010). Interfamily transfer of a plant pattern-recognition receptor confers broad-spectrum bacterial resistance. Nat. Biotechnol. 28, 365–369. 10.1038/nbt.1613 20231819

[B101] Lamesch P. Berardini T. Z. Li D. Swarbreck D. Wilks C. Sasidharan R.. (2011). The Arabidopsis Information Resource (TAIR): improved gene annotation and new tools. Nucleic Acids Res. 40, D1202–D1210. 10.1093/nar/gkr1090 22140109 PMC3245047

[B102] Laquitaine L. Gomès E. François J. Marchive C. Pascal S. Hamdi S.. (2006). Molecular basis of ergosterol-induced protection of grape against *Botrytis cinerea*: induction of type I LTP promoter activity, WRKY, and stilbene synthase gene expression. Mol. Plant Microbe Interact. 19, 1103–1112. 10.1094/MPMI-19-1103 17022174

[B103] Lee J. Klessig D. F. Nürnberger T. (2001). A harpin binding site in tobacco plasma membranes mediates activation of the pathogenesis-related gene *HIN1* independent of extracellular calcium but dependent on mitogen-activated protein kinase activity. Plant Cell 13, 1079–1093. 10.1105/tpc.13.5.1079 11340183 PMC135567

[B104] Li W. Shao M. Zhong W. Yang J. Okada K. Yamane H.. (2012). Ectopic expression of *Hrf1* enhances bacterial resistance via regulation of diterpene phytoalexins, silicon and reactive oxygen species burst in rice. PLoS ONE 7:e43914. 10.1371/journal.pone.0043914 22970151 PMC3435380

[B105] Libault M. Wan J. Czechowski T. Udvardi M. Stacey G. (2007). Identification of 118 *Arabidopsis* transcription factor and 30 ubiquitin-ligase genes responding to chitin, a plant-defense elicitor. Mol. Plant Microbe Interact. 20, 900–911. 10.1094/MPMI-20-8-0900 17722694

[B106] Liebrand T. W. H. van den Berg G. C. M. Zhang Z. Smit P. Cordewener J. H. G. America A. H. P.. (2013). Receptor-like kinase SOBIR1/EVR interacts with receptor-like proteins in plant immunity against fungal infection. Proc. Natl. Acad. Sci. U.S.A. 110, 10010–10015. 10.1073/pnas.1220015110 23716655 PMC3683720

[B107] Liu T. Liu Z. Song C. Hu Y. Han Z. She J.. (2012). Chitin-induced dimerization activates a plant immune receptor. Science 336, 1160–1164. 10.1126/science.1218867 22654057

[B108] Liu W. Liu J. Ning Y. Ding B. Wang X. Wang Z.. (2013). Recent progress in understanding PAMP- and effector-triggered immunity against the rice blast fungus *Magnaporthe oryzae*. Mol. Plant 6, 605–620. 10.1093/mp/sst015 23340743

[B109] Livaja M. Palmieri M. von Rad U. Durner J. (2008a). The effect of the bacterial effector protein harpin on transcriptional profile and mitochondrial proteins of *Arabidopsis thaliana*. J. Proteomics 71, 148–159. 10.1016/j.jprot.2008.04.002 18617142

[B110] Livaja M. Zeidler D. von Rad U. Durner J. (2008b). Transcriptional responses of *Arabidopsis thaliana* to the bacteria-derived PAMPs harpin and lipopolysaccharide. Immunobiology 213, 161–171. 10.1016/j.imbio.2007.10.004 18406364

[B111] Lochman J. Mikes V. (2006). Ergosterol treatment leads to the expression of a specific set of defence-related genes in tobacco. Plant Mol. Biol. 62, 43–51. 10.1007/s11103-006-9002-5 16900324

[B112] Maffei M. E. Arimura G. I. Mithoefer A. (2012). Natural elicitors, effectors and modulators of plant responses. Nat. Prod. Rep. 29, 1288–1303. 10.1039/c2np20053h 22918379

[B113] Mateos F. V. Rickauer M. Esquerré-Tugayé M.-T. (1997). Cloning and characterization of a cDNA encoding an elicitor of *Phytophthora parasitica* var. *nicotianae* that shows cellulose-binding and lectin-like activities. Mol. Plant Microbe Interact. 10, 1045–1053. 10.1094/MPMI.1997.10.9.1045 9390419

[B114] Mazzotta S. Kemmerling B. (2011). Pattern recognition in plant innate immunity. J. Plant Pathol. 93, 7–17.

[B115] McLellan H. Boevink P. C. Armstrong M. R. Pritchard L. Gomez S. Morales J.. (2013). An RxLR effector from *Phytophthora infestans* prevents re-localisation of two plant NAC transcription factors from the endoplasmic reticulum to the nucleus. PLoS Pathog. 9:e1003670. 10.1371/journal.ppat.1003670 24130484 PMC3795001

[B116] Medeiros F. Resende M. Medeiros F. Zhang H. Pare P. (2009). Defense gene expression induced by a coffee-leaf extract formulation in tomato. Physiol. Mol. Plant P. 74, 175–183 10.1016/j.pmpp.2009.11.004

[B117] Mercier L. Lafitte C. Borderies G. Briand X. Esquerré-Tugayé M.-T. Fournier J. (2001). The algal polysaccharide carrageenans can act as an elicitor of plant defence. New Phytol. 149, 43–51 10.1046/j.1469-8137.2001.00011.x 33853239

[B118] Milling A. Babujee L. Allen C. (2011). *Ralstonia solanacearum* extracellular polysaccharide is a specific elicitor of defense responses in wilt-resistant tomato plants. PLoS ONE 6:e15853. 10.1371/journal.pone.0015853 21253019 PMC3017055

[B119] Mizoguchi T. Ichimura K. Irie K. Morris P. Giraudat J. Matsumoto K.. (1998). Identification of a possible MAP kinase cascade in *Arabidopsis thaliana* based on pairwise yeast two-hybrid analysis and functional complementation tests of yeast mutants. FEBS Lett. 437, 56–60. 10.1016/S0014-5793(98)01197-1 9804171

[B120] Mukhtar M. S. Carvunis A. R. Dreze M. Epple P. Steinbrenner J. Moore J.. (2011). Independently evolved virulence effectors converge onto hubs in a plant immune system network. Science 333, 596–601. 10.1126/science.1203659 21798943 PMC3170753

[B121] Newman M. A. Sundelin T. Nielsen J. T. Erbs G. (2013). MAMP (microbe-associated molecular pattern) triggered immunity in plants. Front. Plant Sci. 4:139. 10.3389/fpls.2013.00139 23720666 PMC3655273

[B122] Newton A. C. Fitt B. D. L. Atkins S. D. Walters D. R. Daniell T. J. (2010). Pathogenesis, parasitism and mutualism in the trophic space of microbeplant interactions. Trends Microbiol. 18, 365–373. 10.1016/j.tim.2010.06.002 20598545

[B123] Nguyen H. P. Chakravarthy S. Velásquez A. C. Mclane H. L. Zeng L. R. Nakayashiki H.. (2010). Methods to study PAMP-triggered immunity using tomato and Nicotiana benthamiana. Mol. Plant Microbe Interact. 23, 991–999. 10.1094/MPMI-23-8-0991 20615110

[B124] Nürnberger T. Nennstiel D. Jabs T. Sacks W. R. Hahlbrock K. Scheel D. (1994). High affinity binding of a fungal oligopeptide elicitor to parsley plasma membranes triggers multiple defense responses. Cell 78, 449–460. 10.1016/0092-8674(94)90423-5 8062387

[B125] Parker J. E. (2003). Plant recognition of microbial patterns. Trends Plant Sci. 8, 245–247. 10.1016/S1360-1385(03)00105-5 12818655

[B126] Pastor V. Luna E. Mauch-Mani B. Ton J. Flors V. (2013). Primed plants do not forget. Environ. Exp. Bot. 94, 46–56 10.1016/j.envexpbot.2012.02.013

[B127] Pedersen C. van Themaat E. V. L. McGuffin L. J. Abbott J. C. Burgis T. A. Barton G.. (2012). Structure and evolution of barley powdery mildew effector candidates. BMC Genomics 13:694. 10.1186/1471-2164-13-694 23231440 PMC3582587

[B128] Peng D. H. Qiu D. W. Ruan L. F. Zhou C. F. Sun M. (2011). Protein elicitor PemG1 from *Magnaporthe grisea* induces systemic acquired resistance (SAR) in plants. Mol. Plant Microbe Interact. 24, 1239–1246. 10.1094/MPMI-01-11-0003 21770770

[B129] Povero G. Loreti E. Pucciariello C. Santaniello A. Di Tommaso D. Di Tommaso G.. (2011). Transcript profiling of chitosan-treated Arabidopsis seedlings. J. Plant Res. 124, 619–629. 10.1007/s10265-010-0399-1 21240536

[B130] Po-Wen C. Singh P. Zimmerli L. (2013). Priming of the Arabidopsis pattern-triggered immunity response upon infection by necrotrophic *Pectobacterium carotovorum* bacteria. Mol. Plant Pathol. 14, 58–70. 10.1111/j.1364-3703.2012.00827.x 22947164 PMC6638802

[B131] Qiao Y. Liu L. Xiong Q. Flores C. Wong J. Shi J.. (2013). Oomycete pathogens encode silencing suppressors. Nat. Genet. 45, 330–333. 10.1038/ng.2525 23377181 PMC4049077

[B132] Qiu D. Mao J. Yang X. Zeng H. (2009). Expression of an elicitor-encoding gene from *Magnaporthe grisea* enhances resistance against blast disease in transgenic rice. Plant Cell Rep. 28, 925–933. 10.1007/s00299-009-0698-y 19337737

[B133] Qutob D. Kemmerling B. Brunner F. Küfner I. Engelhardt S. Gust A. A.. (2006). Phytotoxicity and innate immune responses induced by Nep1-like proteins. Plant Cell 18, 3721–3744. 10.1105/tpc.106.044180 17194768 PMC1785393

[B134] Rafiqi M. Ellis J. G. Ludowici V. A. Hardham A. R. Dodds P. N. (2012). Challenges and progress towards understanding the role of effectors in plant-fungal interactions. Curr. Opin. Plant Biol. 15, 477–482. 10.1016/j.pbi.2012.05.003 22658704

[B135] Randoux B. Renard-Merlier D. Mulard G. Rossard S. Duyme F. Sanssené J.. (2010). Distinct defenses induced in wheat against powdery mildew by acetylated and nonacetylated oligogalacturonides. Phytopathology 100, 1352–1363. 10.1094/PHYTO-03-10-0086 20684658

[B136] Reglinski T. Lyon G. D. Newton A. C. (1994a). Assessment of the ability of yeast-derived elicitors to control barley powdery mildew in the field. Z. Pflanzenk. Pflanzen. 101, 1–10.

[B137] Reglinski T. Lyon G. D. Newton A. C. (1994b). Induction of resistance mechanisms in barley by yeast-derived elicitors. Ann. Appl. Biol. 124, 509–517 10.1111/j.1744-7348.1994.tb04155.x

[B138] Reglinski T. Lyon G. D. Newton A. C. (1995). The control of *Botrytis cinerea* and *Rhizoctonia solani* on lettuce using elicitors extracted from yeast cell walls. Z. Pflanzenk. Pflanzen. 102, 257–266.

[B139] Ron M. Avni A. (2004). The receptor for the fungal elicitor ethylene-inducing xylanase is a member of a resistance-like gene family in tomato. Plant Cell 16, 1604–1615. 10.1105/tpc.022475 15155877 PMC490049

[B140] Sangha J. S. Ravichandran S. Prithiviraj K. Critchley A. T. Prithiviraj B. (2010). Sulfated macroalgal polysaccharides lambda-carrageenan and iota-carrageenan differentially alter *Arabidopsis thaliana* resistance to *Sclerotinia sclerotiorum*. Physiol. Mol. Plant P. 75, 38–45 10.1016/j.pmpp.2010.08.003

[B141] Schönbeck F. Dehne H.-W. Balder H. (1982). Zur Wirksamkeit induzierter Resistenz unter praktischen Anbaubedingungen I. Echter Mehltau an Reben, Gurken und Weizen. Z. Pflanzenk. Pflanzen. 89, 177–184.

[B142] Schönbeck F. Dehne H.-W. Beicht W. (1980). Untersuchungen zur Aktivierung unspeziefischer Resistenzmechanismen in Pflanzen. Z. Pflanzenk. Pflanzen. 87, 654–666.

[B143] Schornack S. Huitema E. Cano L. M. Bozkurt T. O. Oliva R. Van Damme M.. (2009). Ten things to know about oomycete effectors. Mol. Plant Pathol. 10, 795–803. 10.1111/j.1364-3703.2009.00593.x 19849785 PMC6640533

[B144] Shimizu T. Nakano T. Takamizawa D. Desaki Y. Ishii-Minami N. Nishizawa Y.. (2010). Two LysM receptor molecules, CEBiP and OsCERK1, cooperatively regulate chitin elicitor signaling in rice. Plant J. 64, 204–214. 10.1111/j.1365-313X.2010.04324.x 21070404 PMC2996852

[B145] Shinya T. Gális I. Narisawa T. Sasaki M. Fukuda H. Matsuoka H.. (2007). Comprehensive analysis of glucan elicitor-regulated gene expression in tobacco BY-2 cells reveals a novel MYB transcription factor involved in the regulation of phenylpropanoid metabolism. Plant Cell Physiol. 48, 1404–1413. 10.1093/pcp/pcm115 17761750

[B146] Shinya T. Motoyama N. Ikeda A. Wada M. Kamiya K. Hayafune M.. (2012). Functional characterization of CEBiP and CERK1 homologs in Arabidopsis and rice reveals the presence of different chitin receptor systems in plants. Plant Cell Physiol. 53, 1696–1706. 10.1093/pcp/pcs113 22891159

[B147] Shoresh M. Yedidia I. Chet I. (2005). Involvement of jasmonic acid/ethylene signaling pathway in the systemic resistance induced in cucumber by *Trichoderma asperellum* T203. Phytopathology 95, 76–84. 10.1094/PHYTO-95-0076 18943839

[B148] Steiner U. Oerke E.-C. Schönbeck F. (1988). Zur Wirksamkeit der induzierten Resistenz unter praktischen Anbaubedingungen IV. Befall und Ertrag von Wintergerstensorten mit induzierter Resistenz und nach Fungizidbehandlung. Z. Pflanzenk. Pflanzen. 95, 506–517. 21395787

[B149] Suzuki H. Reddy M. S. S. Naoumkina M. Aziz N. May G. D. Huhman D. V.. (2005). Methyl jasmonate and yeast elicitor induce differential transcriptional and metabolic re-programming in cell suspension cultures of the model legume *Medicago truncatula*. Planta 220, 696–707. 10.1007/s00425-004-1387-2 15605242

[B150] Takahashi Y. Nasir K. H. B. Ito A. Kanzaki H. Matsumura H. Saitoh H. (2007). A high-throughput screen of cell-death-inducing factors in *Nicotiana benthamiana* identifies a novel MAPKK that mediates INF1-induced cell death signaling and non-host resistance to *Pseudomonas cichorii*. Plant J. 49, 1030–1040 10.1111/j.1365-313X.2006.03022.x 17319846

[B151] Takken F. L. W. Luderer R. Gabriëls S. H. E. J. Westerink N. Lu R. De Wit P. J. G. M.. (2000). A functional cloning strategy, based on a binary PVX-expression vector, to isolate HR-inducing cDNAs of plant pathogens. Plant J. 24, 275–283. 10.1046/j.1365-313x.2000.00866.x 11069701

[B152] Tanaka S. Ichikawa A. Yamada K. Tsuji G. Nishiuchi T. Mori M.. (2010). *HvCEBiP*, a gene homologous to rice chitin receptor *CEBiP*, contributes to basal resistance of barley to *Magnaporthe oryzae*. BMC Plant Biol. 10:288. 10.1186/1471-2229-10-288 21190588 PMC3020183

[B153] Thomas C. M. Jones D. A. Parniske M. Harrison K. Balint-Kurti P. J. Hatzixanthis K.. (1997). Characterization of the tomato *Cf-4* gene for resistance to *Cladosporium fulvum* identifies sequences that determine recognitional specificity in Cf-4 and Cf-9. Plant Cell 9, 2209–2224. 10.1105/tpc.9.12.2209 9437864 PMC157069

[B154] Umemura K. Tanino S. Nagatsuka T. Koga J. Iwata M. Nagashima K.. (2004). Cerebroside elicitor confers resistance to Fusarium disease in various plant species. Phytopathology 94, 813–818. 10.1094/PHYTO.2004.94.8.813 18943100

[B155] Underwood W. (2012). The plant cell wall: a dynamic barrier against pathogen invasion. Front. Plant Sci. 3:85. 10.3389/fpls.2012.00085 22639669 PMC3355688

[B156] van den Burg H. A. Harrison S. J. Joosten M. H. A. J. Vervoort J. De Wit P. J. G. M. (2006). *Cladosporium fulvum* Avr4 protects fungal cell walls against hydrolysis by plant chitinases accumulating during infection. Mol. Plant Microbe Interact. 19, 1420–1430. 10.1094/MPMI-19-1420 17153926

[B157] van Hulten M. Pelser M. van Loon L. C. Pieterse C. M. J. Ton J. (2006). Costs and benefits of priming for defense in *Arabidopsis*. Proc. Natl. Acad. Sci. U.S.A. 103, 5602–5607. 10.1073/pnas.0510213103 16565218 PMC1459400

[B158] Vatsa P. Chiltz A. Luini E. Vandelle E. Pugin A. Roblin G. (2011). Cytosolic calcium rises and related events in ergosterol-treated *Nicotiana* cells. Plant Physiol. Biochem. 49, 764–773. 10.1016/j.plaphy.2011.04.002 21530285

[B159] Vera J. Castro J. Gonzalez A. Moenne A. (2011). Seaweed polysaccharides and derived oligosaccharides stimulate defense responses and protection against pathogens in plants. Mar. Drugs 9, 2514–2525. 10.3390/md9122514 22363237 PMC3280573

[B160] Viterbo A. D. A. Wiest A. R. I. C. Brotman Y. A. R. I. Chet I. L. A. N. Kenerley C. H. A. R. (2007). The 18mer peptaibols from *Trichoderma virens* elicit plant defence responses. Mol. Plant Pathol. 8, 737–746. 10.1111/j.1364-3703.2007.00430.x 20507534

[B161] Walters D. Bingham I. (2007). Influence of nutrition on disease development caused by fungal pathogens: implications for plant disease control. Ann. Appl. Biol. 151, 307–324 10.1111/j.1744-7348.2007.00176.x

[B162] Walters D. Heil M. (2007). Costs and trade-offs associated with induced resistance. Physiol. Mol. Plant P. 71, 3–17 10.1016/j.pmpp.2007.09.008

[B163] Walters D. R. Ratsep J. Havis N. D. (2013). Controlling crop diseases using induced resistance: challenges for the future. J. Exp. Bot. 64, 1263–1280. 10.1093/jxb/ert026 23386685

[B164] Wan J. Zhang X. C. Neece D. Ramonell K. M. Clough S. Kim S.. (2008). A LysM receptor-like kinase plays a critical role in chitin signaling and fungal resistance in *Arabidopsis*. Plant Cell 20, 471–481. 10.1105/tpc.107.056754 18263776 PMC2276435

[B165] Wang B. Wang S. Tan B. Qiu D. Yang X. (2012a). Systemic acquired resistance to Tobacco mosaic virus (TMV) induced by protein elicitor from *Verticillium dahliae* (PevD1) and its mechanisms in tobacco. J. Agr. Biotechnol. 20, 188–195. 21691787

[B166] Wang B. Yang X. Zeng H. Liu H. Zhou T. Tan B.. (2012b). The purification and characterization of a novel hypersensitive-like response-inducing elicitor from *Verticillium dahliae* that induces resistance responses in tobacco. App. Microbiol. Biotechnol. 93, 191–201. 10.1007/s00253-011-3405-1 21691787

[B167] Wang F. Feng G. Chen K. (2009). Burdock fructooligosaccharide induces resistance to tobacco mosaic virus in tobacco seedlings. Physiol. Mol. Plant P. 74, 34–40 10.1016/j.pmpp.2009.08.002

[B168] Wang S. Durrant W. E. Song J. Spivey N. W. Dong X. (2010). *Arabidopsis* BRCA2 and RAD51 proteins are specifically involved in defense gene transcription during plant immune responses. Proc. Natl. Acad. Sci. U.S.A. 107, 22716–22721. 10.1073/pnas.1005978107 21149701 PMC3012525

[B169] Wei H. Xu Q. Taylor L. E. 2nd. Baker J. O. Tucker M. P. Ding S. Y. (2009). Natural paradigms of plant cell wall degradation. Curr. Opin. Biotechnol. 20, 330–338. 10.1016/j.copbio.2009.05.008 19523812

[B170] Xu Y. Chen H. Zhou X. Cai X. (2012). Induction of hypersensitive response and nonhost resistance by a *Cladosporium fulvum* elicitor CfHNNI1 is dose-dependent and negatively regulated by salicylic acid. J. Integr. Agri. 11, 1665–1674 10.1016/S2095-3119(12)60169-5

[B171] Yang D. H. Hettenhausen C. Baldwin I. T. Wu J. (2011). The multifaceted function of BAK1/SERK3: plant immunity to pathogens and responses to insect herbivores. Plant Signal. Behav. 6, 1322–1324. 10.4161/psb.6.9.16438 21852758 PMC3258060

[B172] Yang Y. Zhang H. Li G. Li W. Wang X. Song F. (2009). Ectopic expression of MgSM1, a Cerato-platanin family protein from *Magnaporthe grisea*, confers broad-spectrum disease resistance in Arabidopsis. Plant Biotechnol. J. 7, 763–777. 10.1111/j.1467-7652.2009.00442.x 19754836

[B173] Zhang J. Shao F. Li Y. Cui H. Chen L. Li H.. (2007). A *Pseudomonas syringae* effector inactivates MAPKs to suppress PAMP-induced immunity in plants. Cell Host Microbe 1, 175–185. 10.1016/j.chom.2007.03.006 18005697

[B174] Zhang J. Tong Z. Gao Z. Luo C. Qu S. Zhang Z. (2011a). Expression of MhWRKY1 gene induced by the elicitors SA, MeJA, ACC and the apple ring spot pathogen. Sci. Agric. Sin. 44, 990–999.

[B175] Zhang W. Yang X. Qiu D. Guo L. Zeng H. Mao J.. (2011b). PeaT1-induced systemic acquired resistance in tobacco follows salicylic acid-dependent pathway. Mol. Biol. Rep. 38, 2549–2556. 10.1007/s11033-010-0393-7 21088909

[B176] Zhang W. Fraiture M. Kolb D. Löffelhardt B. Desaki Y. Boutrot F. F.. (2013). *Arabidopsis* receptor-like protein30 and receptor-like kinase suppressor of BIR1-1/EVERSHED mediate innate immunity to necrotrophic fungi. Plant Cell 25, 4227–4241. 10.1105/tpc.113.117010 24104566 PMC3877809

[B177] Zhang Y. Yang X. Liu Q. Qiu D. Zhang Y. Zeng H.. (2010). Purification of novel protein elicitor from *Botrytis cinerea* that induces disease resistance and drought tolerance in plants. Microbiol. Res. 165, 142–151. 10.1016/j.micres.2009.03.004 19616421

[B178] Zheng X. McLellan H. Fraiture M. Liu X. Boevink P. C. Gilroy E. M.. (2014). Functionally redundant RXLR effectors from *Phytophthora infestans* act at different steps to suppress early flg22-triggered immunity. PLoS Pathog. 10:e1004057. 10.1371/journal.ppat.1004057 24763622 PMC3999189

[B179] Zipfel C. Kunze G. Chinchilla D. Caniard A. Jones J. D. G. Boller T.. (2006). Perception of the bacterial PAMP EF-Tu by the receptor EFR restricts *Agrobacterium*-mediated transformation. Cell 125, 749–760. 10.1016/j.cell.2006.03.037 16713565

[B180] Zipfel C. Robatzek S. Navarro L. Oakeley E. J. Jones J. D. G. Felix G.. (2004). Bacterial disease resistance in *Arabidopsis* through flagellin perception. Nature 428, 764–767. 10.1038/nature02485 15085136

