# Supplementary material for: Molecular effects of resistance elicitors from biological origin and their potential for crop protection
Source: Front Plant Sci. 2014 Nov 21;5:655. doi: 10.3389/fpls.2014.00655 (PMC4240061; doi:10.3389/fpls.2014.00655)
Supplement: Supplementary file 1 [file Presentation1.PDF]

Supplementary material

Molecular effects of resistance elicitors of biological origins: uncoupling host recognition responses from pathogen defence suppression.

Lea Wiesel[1], Adrian C. Newton[1], Ian Elliott[2], David Booty[2], Eleanor M. Gilroy[1], Paul R. J. Birch[1,3], Ingo Hein[1,*]

[1]The James Hutton Institute, Invergowrie, Dundee, UK
[2]OMEX Agriculture Ltd, Bardney Airfield, Tupholme, Lincoln, UK
[3]The Division of Plant Sciences, College of Life Science, University of Dundee at the James Hutton Institute, Invergowrie, Dundee, UK

[*]Correspondence:
Ingo Hein
The James Hutton Institute
Invergowrie
Dundee
DD2 5DA
UK
ingo.hein@hutton.ac.uk
+448449285428

Supplementary figures:

Supplementary Figure S1: Hierarchical visualisation of elicitor induced genes that are over-represented in the GO term ‘biological processes’. Inside the box of the significant terms, the information includes: GO term, adjusted p-value, GO description, item number mapping the GO in the query list and background and total number of query list and background. The darker and redder the colour of the box, the smaller and more significant is the p-value. Stars highlight GO terms that contain genes targeted by pathogen effectors.

Supplementary Figure S2: Hierarchical visualisation of elicitor induced genes that are over-represented in the GO term ‘molecular functions’. Inside the box of the significant terms, the information includes: GO term, adjusted p-value, GO description, item number mapping the GO in the query list and background, and total number of query list and background. The darker and redder the colour of the box, the smaller and more significant is the p-value. Stars highlight GO terms that contain genes targeted by pathogen effectors.

Supplementary Figure S3: Hierarchical visualisation of elicitor induced genes that are over-represented in the GO term ‘cell parts’. Inside the box of the significant terms, the information includes: GO term, adjusted p-value, GO description, item number mapping the GO in the query list and background, and total number of query list and background. The darker and redder the colour of the box, the smaller and more significant is the p-value. Stars highlight GO terms that contain genes targeted by pathogen effectors.

Supplementary tables:

Supplementary Table S1: *Arabidopsis thaliana* genes that have been shown to be activated by the recognition of elicitor compounds (S1A) and associated publications in alphabetical order (S1B).

Supplementary Table S2: Significant shared GO terms determined by agriGo (Du et al., 2010) using *Arabidopsis thaliana* genes that have been shown to be activated by the detection of elicitor compounds. Term-type P stands for biological process, F for molecular function and C for cellular component. Queryitem gives the number of genes of interest within the GO term, querytotal the total number of genes in the list of interest. Bgitem gives the number of background genes within the GO term, bgtotal the total number of genes in the genome.

Supplementary Table S3: *Arabidopsis thaliana* genes upregulated upon elicitor recognition and also targeted by pathogen effectors. For each gene, description and annotation by TAIR and involvement in GO-terms are listed.
