# Supplementary figures and images for: Molecular effects of resistance elicitors from biological origin and their potential for crop protection
Source: Front Plant Sci. 2014 Nov 21;5:655. doi: 10.3389/fpls.2014.00655 (PMC4240061; doi:10.3389/fpls.2014.00655)

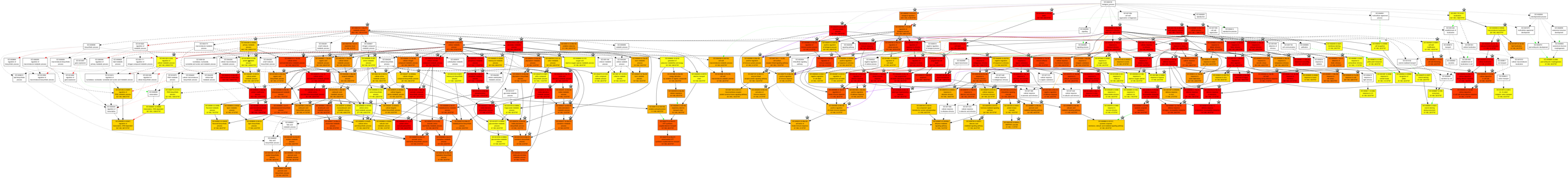

Supplement: Supplementary file 5 [file Image1.TIF]

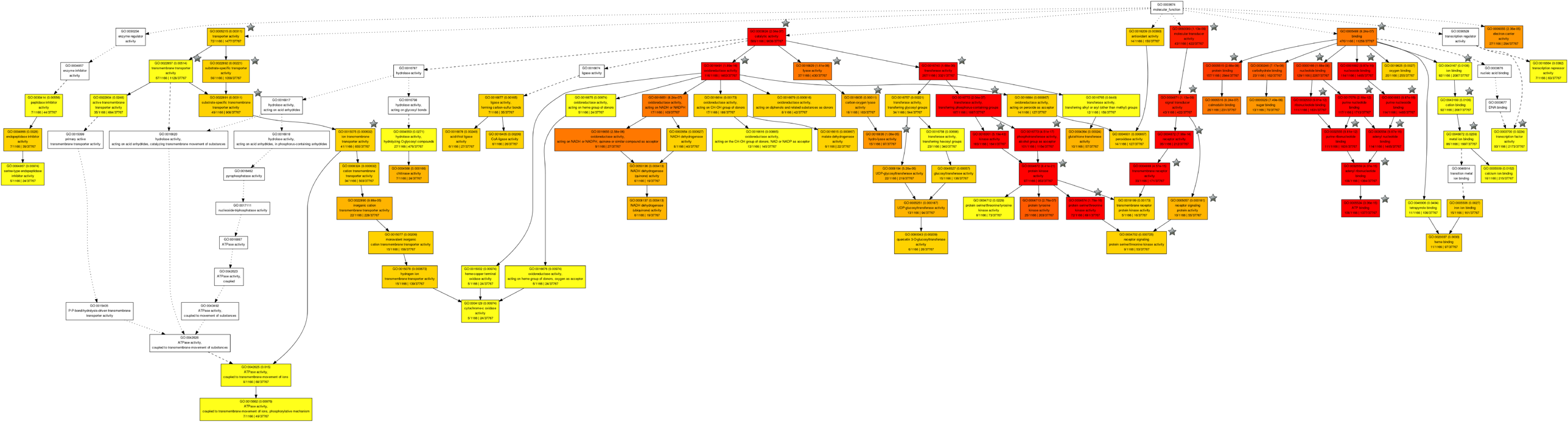

Supplement: Supplementary file 6 [file Image2.TIF]

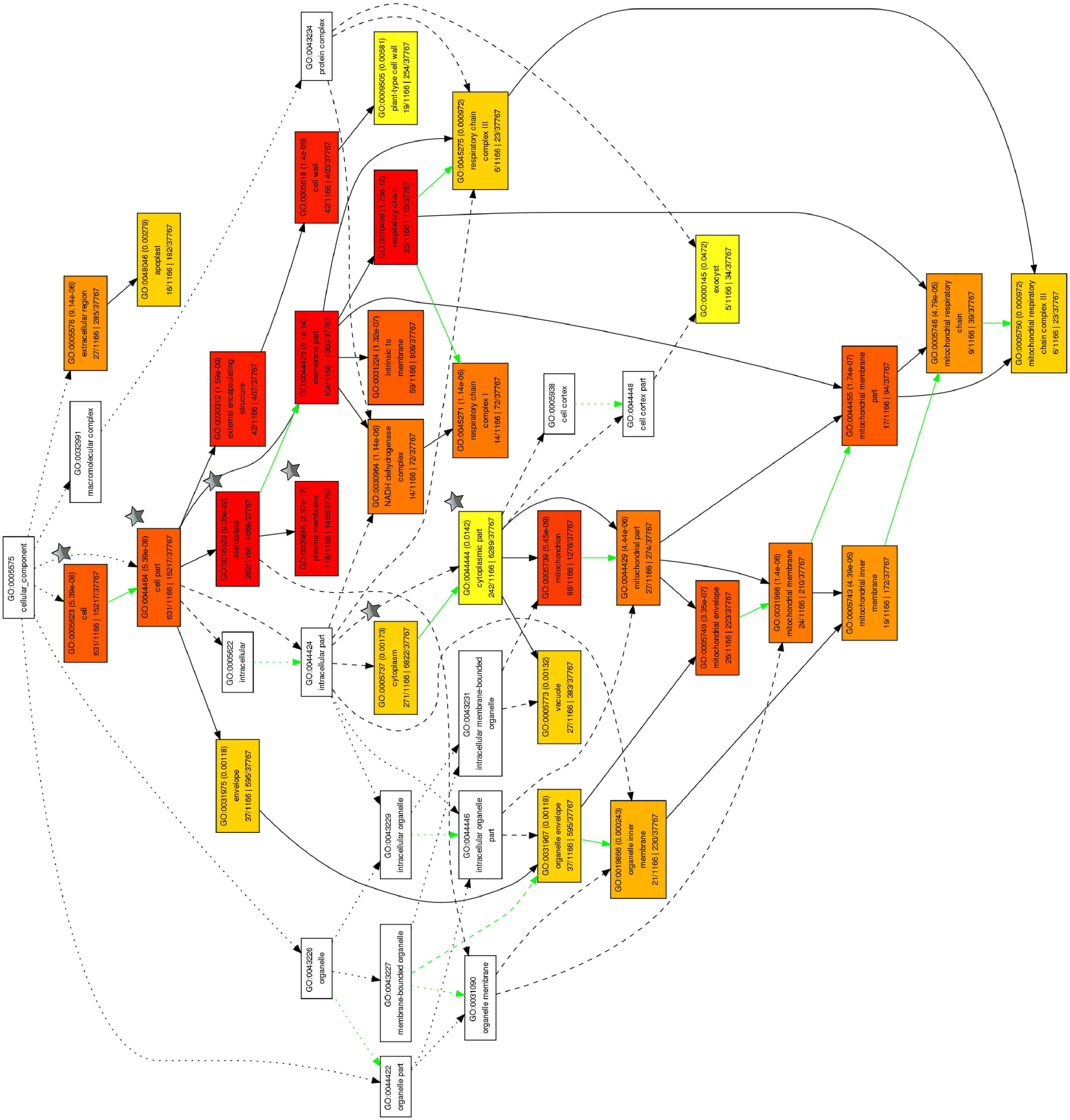

Supplement: Supplementary file 7 [file Image3.TIF]
